# A holistic stochastic model for precipitation events

**DOI:** 10.1038/s41598-024-77031-3

**Published:** 2025-02-07

**Authors:** Alexander Weyant, Alexander Gershunov, Anna K. Panorska, Tomasz J. Kozubowski, Julie Kalansky

**Affiliations:** 1 Scripps Institution of Oceanography, Climate, Atmospheric Sciences, and Physical Oceanography Division, La Jolla, CA 92037 USA; 2 Department of Mathematics and Statistics, University of Nevada, Reno, NV 89557 USA

**Keywords:** Extreme precipitation, Return periods, Return intervals, Peaks-over-threshold, Climate and Earth system modelling, Hydrology, Climate sciences

## Abstract

In the western United States, much of the annual precipitation falls during relatively few storm events. When precipitation is measured as daily (or hourly, etc.) accumulations, these events appear as sequences of various durations. We describe a trivariate probability distribution and assert that it provides a natural description for the durations, magnitudes, and maximum intensities of such events. We show that this distribution, in its most straightforward application, indeed describes precipitation events composed of daily observations at most long-term weather stations across the western United States, allowing for a flexible assessment of specific event probabilities and return periods with respect to their three defining components. This characterization opens the door to further understanding of risk associated with out-of-sample meteorological events.

## Introduction

The ability to realistically assess probabilities and return periods of extreme meteorological and hydrological events is important for numerous critical applications ranging from engineering design limits to impact and liability assessments. Extreme temperature, wind, precipitation, and streamflow events impact various sectors of society from infrastructure to public health, agriculture, and energy supply and demand. For most weather extremes of interest, probability models are often fitted to some extreme manifestation of an observed time series (e.g. maximum value of hourly or daily precipitation) representing the highest intensity observation per block (e.g. season or year). The probability model is then used to estimate return periods of extremes, including out-of-sample occurrences. This approach is referred to as the Block Maxima approach to Extreme Value (EV) modeling^1^. A prominent example of the application of EV modeling to precipitation extremes is the National Oceanic and Atmospheric Administration (NOAA) Precipitation-Frequency Atlas 14^2^, which provides a set of location-specific tables matching precipitation accumulations over a set of prescribed periods (e.g. hourly, daily, etc.) with expected frequencies expressed as return periods.

An alternative probabilistic approach for heavy precipitation modeling is the Peaks-Over-Threshold (POT) framework, where values truncated above a high threshold (e.g. 90th or 95th percentile of local non-zero observations), or “Exceedances”, become the relevant data points^1^. The statistical basis of the POT modeling is the Balkema–Pickands–de Haan theorem^3,4^ which specifies the Generalized Pareto distribution for the Exceedances asymptotically for increasing thresholds. The POT method yields return periods for the extreme events understood as Exceedances of hourly/daily/monthly observations over a high threshold, which are used in a similar way to those derived with the Block Maxima approach. One advantage POT methods have over GEV/Block Maxima is that they are fitted to more data and so can represent all heavy, including extreme, precipitation, rather than just the most extreme values in a block. POT models can also incorporate more data from physical events, such as individual storms, but they very often (with rare exceptions^5^) neglect an important aspect of events: *namely, that their durations vary*. The same is true of intensity-duration-frequency (IDF) analysis, which provides an internally consistent assessment of the probability of precipitation accumulations over *prescribed* time intervals^6^. IDF analysis, along with all aforementioned approaches, provide solutions to problems distinct from the study of events of random durations.

**Fig. 1:**
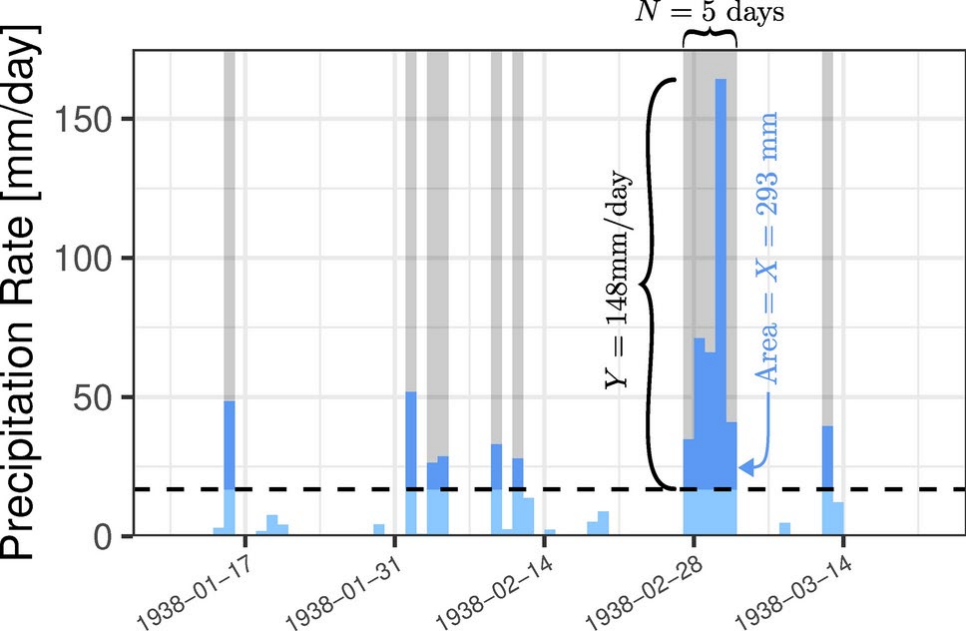
A schematic of how events are defined and identified in a series of daily precipitation accumulations at San Gabriel Canyon Pumphouse, CA; Accumulations above a threshold are shown in dark blue. Seven separate Events are visible, each with its own duration (width of grey shaded region), maximum intensity (height of dark blue region), and magnitude (area of the dark blue region).

In order to statistically represent physical events such as storms, more holistic models that describe several of their characteristics *simultaneously* are required. To this end, we propose a trivariate probability model describing (variable) duration, maximum intensity and overall magnitude of events, such as those depicted in Fig. 1. The events are represented by the sets of consecutive observations above a threshold. Each event is described by three characteristics: duration *N*, maximum intensity *Y*, and magnitude *X*, collected into a vector (*X*, *Y*, *N*). Since *X*, *Y* and *N* are all random quantities, we have a random vector model for the events, and we describe it with a trivariate probability distribution.

To make sensible inferences about out-of-sample events, we need to fit a physically-consistent universal model to precipitation event summaries across different locations (e.g. stations) and regions. Although other approaches (e.g. copula-based methods^7–10^) exist to achieve trivariate event modeling of precipitation, these models are not necessarily chosen to be physically meaningful. In practice, the goal of such approaches is to achieve the best possible fit to sample data without regard to the process that produced it. A copula-based mindset and approach presupposes that marginal distributions and their dependence structure can be considered separately. The marginals which apparently provide the best fit to a sample are often chosen without regard for the fact that maxima and sums of dependent components ought to be fundamentally related^11^. For example, it would not make sense for marginal distributions of sums and maxima of the same events to be heavy-tailed and light tailed, respectively. Likewise, it also does not make sense to apply a continuous distribution to decidedly discrete values (e.g. Grimaldi and Serinaldi^7^). Our approach to event modeling is of a different character in that it follows naturally and theoretically from facts about the precipitation data and their treatment:

- Truncation of values over a threshold begs a connection to POT theory. We consider sequences of consecutive Exceedances to be comprised of dependent values from a heavy-tailed (generalized Pareto) distribution, which is consistent with previous work on Exceedances^12–14^.
- Discrete lengths (durations) of sequences of consecutive Exceedances, appropriately, have a discrete distribution.
- The joint distribution of the (*X*, *Y*, *N*) is precisely that which follows theoretically from taking the sum and maximum of such dependent, Pareto (heavy-tailed/outlier-prone) values over a random discrete duration^15^.

Note that, in contrast to a copula approach, a single set of parameters is shared by the entire joint distribution, no matter how it is viewed (integrated, conditioned, or otherwise transformed) and the estimation of parameters takes place in a unified procedure.

We call a model which arises theoretically from the data summarization process (truncating over a threshold, taking the sum and maximum) a proper *TED (trivariate event distribution)*. TEDs have been devised for events and applied to various systems including weather^16,17^ and modeling of its parameters with respect to exogenous covariates can provide insight. However, when a previously introduced exponential case of our present TED^16^ is applied to precipitation Events, it tends to underestimate Event magnitudes. In the “Methods” section, we describe in greater detail how lessons learned about heavy-tails in the distribution of daily precipitation totals^12–14^, a bivariate distribution of sums and maxima of Pareto vectors of prescribed lengths^15^, and a discrete distribution for Event durations^18^ all taken together suggest a new and improved TED for precipitation Events.

Our new TED provides a physically-grounded and universal trivariate model for precipitation events, i.e. meteorological events with random duration. The marginal distribution functions for event maxima and magnitudes arise theoretically as random maxima and random sums, respectively, of Pareto II values dependent within events. The daily values which constitute the Events being Pareto is consistent with the application of POT theory to precipitation^3,14^ and the marginal for duration is an appropriately discrete distribution. Of course, our TED, designed to reflect the properties of meteorological precipitation events, needs to be validated as fitting the data well and this is done below. However, achieving the best possible fit to sample data is not our goal. Our goal is credible inference, which is underpinned by the theoretical and practical requirements of a model that is physically based and consistently applied. Technical aspects of the TED are detailed in “Methods” section below and we highly recommend reading them.

We have applied this new TED to decades-long records of daily precipitation observed at over 4000 individual stations across the western United States (WUS). Precipitation observed on this network reflects a limited variety of regimes and storm types as well the strongly orographic nature of precipitation over this mountainous midlatitude region. After briefly introducing the data and their treatment, we describe the model’s essential parameters and validation results. We then show how the TED can be applied over the WUS and take a closer look at one location in Southern California.

## Data and their treatment

### Observations

We used station data from NOAA’s *Global Historical Climatology Network-Daily* (GHCNd) database^19,20^. It is a vast collection of daily station records from multiple networks: mostly, first-order and *Cooperative Observer Program* (COOP) stations. The observations from different networks are all subjected to a common collection of quality control methods, but they are not homogenized to account for changes over time in instrumentation, standards, and the positioning and surroundings of the rain gauges. We obtained observations from GHCNd stations from the Pacific coast all the way eastward to the states intersected by the 100th meridian west up until January 2023. Very recent observations (such as within the current month) are not always available directly from NOAA. In our case study section, observations since January 2023 were obtained using the Synoptic Data PBC Mesonet API.

At each station, we found how many observations there would be if none were missing. Then, we eliminated observations which had measurement flag “A” (meaning precipitation is actually a multi-day accumulation since the last observation) or quality flags “D” (a sequence of values were probably duplicated from another year or month) or “K” (identical, non-zero values appear in sequence). We counted how many observations remained after this filtering and found the proportion of “valid” observations to the maximum possible number of observations (1 per day). If this proportion at a station was less than 0.95 or if its record was less than 10 years long, we excluded the station from further analysis. Out of the 31,879 stations, 4307 remained after filtering.

### Identifying precipitation events

#### Truncation over a threshold

At most stations, 0.3 mm (0.01 in) was the smallest non-zero precipitation observation. We considered days with precipitation greater than 0.3 mm to be “wet days”. At each station, we found the sample 75th percentile of precipitation observations among all wet days. As in a POT analysis of daily precipitation, we subtract this threshold from precipitation observations and cull all resulting negative values. What remains is a record of precipitation truncated above a threshold, which we call Exceedances.

#### Defining events

We define an Event to be a sequence of consecutive non-zero Exceedances and we summarized these Events in terms of their sums (“magnitudes”), maxima (“maximum intensities”), and lengths (“durations”). Figure 1 shows us examples of precipitation Events observed over one 3-month period—January through March of 1938.

$$\begin{aligned} \textbf{E} \overset{\text {d}}{=} \big (X_{1}, X_{2},\dots , X_{N}\big ) \text { is an Event and } \textbf{T} \overset{\text {d}}{=} \Bigg (\sum _{k=1}^N X_{k}, \bigvee _{k = 1}^N X_{k}, N\Bigg ) \text { is its trivariate summary.} \end{aligned}$$

We study the distribution of trivariate Events summaries, which we merely call “Events” from here on. ($\mathbf {T_i}$) are the same trivariate summaries studied by Kozubowski et al.^16^ and to which a special case of our current TED has been applied^18^.

## Results and discussion

### A parametric summary of all events at each station

**Fig. 2:**
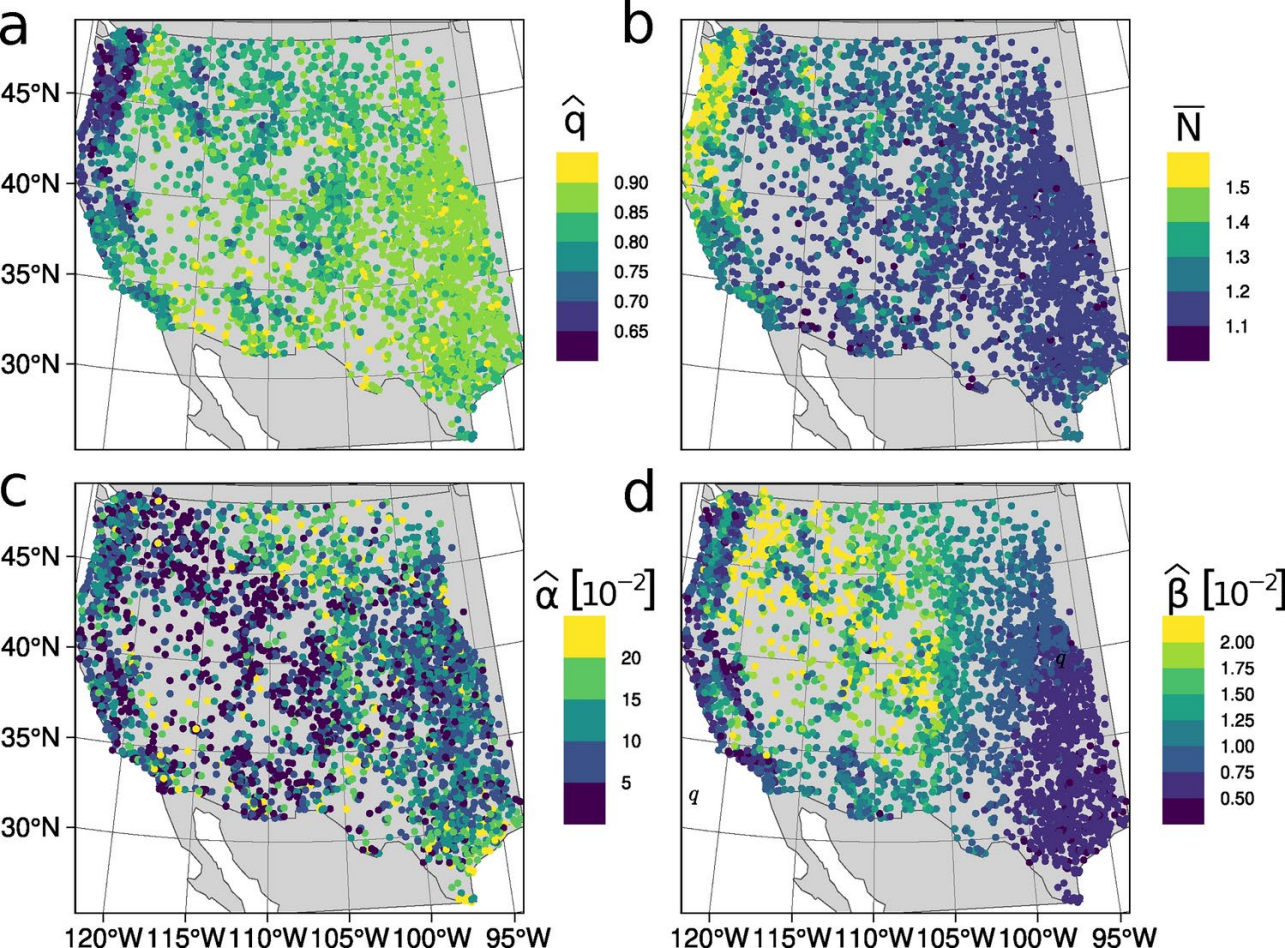
Estimates of three HGSMP parameters and a key derived quantity plotted at GHCNd stations: (**a**) $\widehat{q}$, the estimated probability that an Event only last one day; (**b**) $\overline{N}$, mean Event duration; (**c**) $\widehat{\alpha }$, the estimated tail parameter; (**d**) $\widehat{\beta }$, the estimated scale parameter.

We consider a day to be part of a precipitation Event if it surpasses a certain threshold. In this work, the threshold is specifically defined as the empirical 75th percentile (among wet days) of daily precipitation at each station. Although the thresholds defined at each station can seem quite high when we consider the local climatologies, most precipitation indeed falls during Events. At almost all stations (c.99%), at least half of all recorded precipitation fell during Events. At 94 percent of stations, between 50 and 75% of precipitation was recorded during Events (Supplemental Fig. S10). This is reiteration of a well-known fact about daily precipitation in the WUS (see Dettinger et al.^21^, Fig. 2, for instance) which largely motivates our focus on trivariate Events of precipitation exceeding a high threshold. Since the number of wet days per year varies greatly over the WUS, so does the number of recorded Events per year. Supplemental Figure S10 shows that in most of California and the arid Southwest, 5–15 precipitation events are expected per year. In the Pacific Northwest, events are detected much more frequently, especially farther North. The classic arid-humid dividing meridian of approximately 100° W^22^ is also apparent. Next, we shall physically interpret the TED parameters and see how they reflect the diverse hydroclimates of the region. The parameters’ mathematical description can be found in “Probability distributions” in the “Methods” section.

The west coast, particularly the Pacific Northwest, tends to have longer Events compared to other regions (Fig. 2a,b). This is intuitive, as there are more wet days to combine into Events to begin with and a great proportion of the precipitation comes from synoptic systems (often associated with Atmospheric Rivers (ARs)^23^ and “families” thereof^24^). A large-scale system moving over a weather station for a few days is more likely to manifest as a multi-day Event (at least from our narrow view of daily precipitation crossing a threshold) than a small-scale convective storm on a Summer afternoon. While smaller systems are very likely to occur on consecutive days within moderately-sized regions, they are apparently not very likely to *directly* overlie a given rain gauge on consecutive days, which would be necessary to produce the unbroken runs of very large daily totals.

**Fig. 3:**
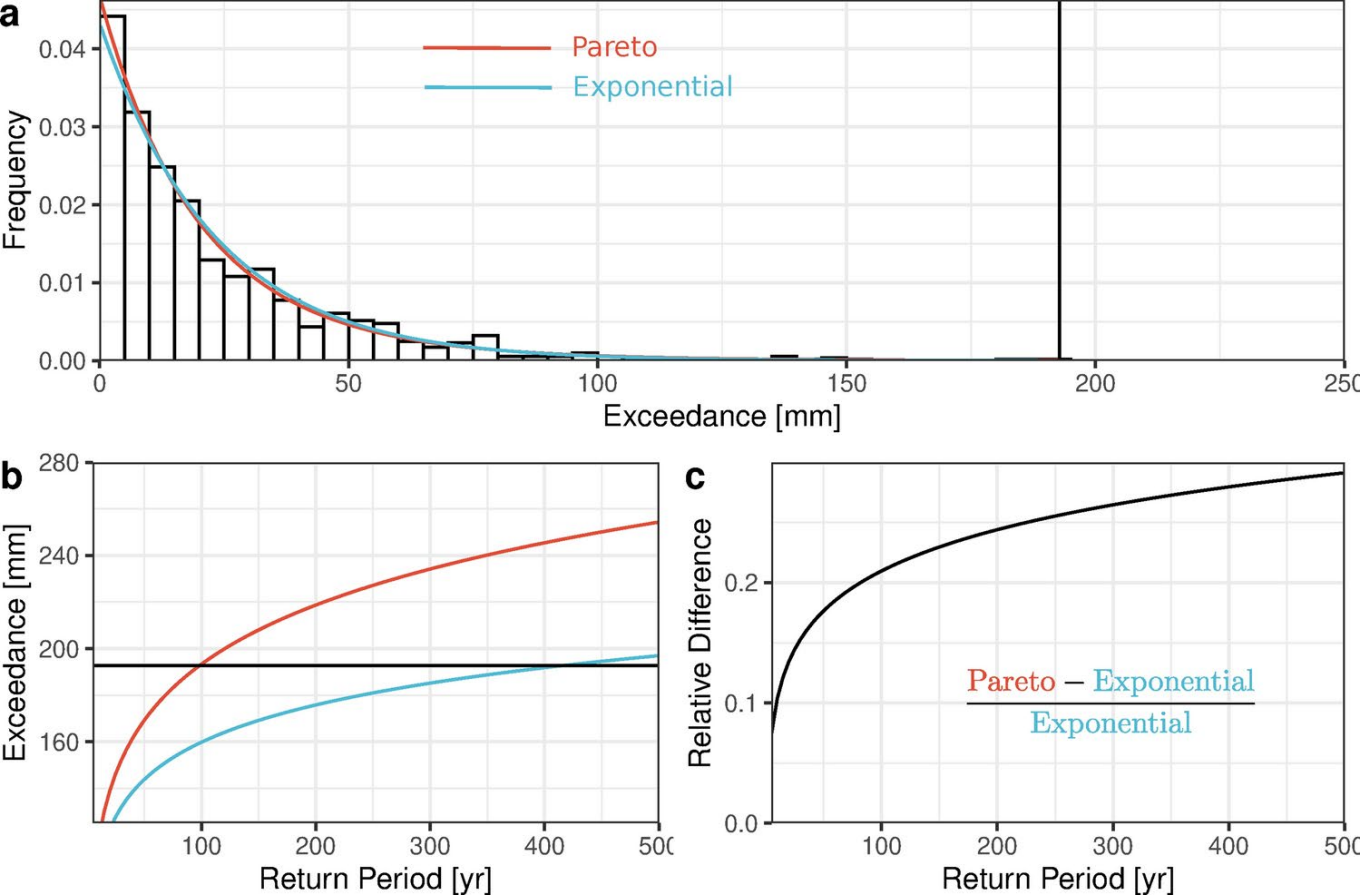
Precipitation Exceedances at San Gabriel Canyon PH from 1 Jan 1917 to 31 December 2021; **(a)** Histogram of all observations, with the largest observation marked with a vertical black line, **(b)** Theoretical return levels calculated from (Univariate) Pareto II and Exponential distributions plotted in red and blue, respectively, **(c)** the same return levels as in (**b**), but expressed as a relative difference of Pareto II and Exponential values.

Multiple interpretations of the tail and scale parameters ($\alpha$ and $\beta$) are provided in “Methods” section, but their simplest (univariate) interpretation is very useful when reading the plot of their estimates in Fig. 2. Any quantile (return level) of the theoretical distribution of Exceedances is proportional to the reciprocal of $\beta$. Therefore, dark spots on Fig. 2d correspond to places with large precipitation Exceedances, making “(reciprocal) scale” an appropriate name for $\beta$. The parameter $\alpha$ tells us to what degree the shape of our distribution of Exceedances differs from an exponential distribution, i.e. the “heaviness” of its probability tail or its propensity for producing “outliers”. The exponential distribution corresponds to the limit as $\alpha$ goes to 0 and specifying this distribution over this entire domain has been shown to lead to severe underestimates of return levels for large precipitation observations at most locations (see Fig. 3 and Panorska et al.^12^).

Given $\alpha$, $\beta$, and a probability, we can directly calculate a theoretical quantile using Eq. (4). A key observation is that given a probability (e.g. $p=0.01$ for a “1/100 Exceedance”) and a scale (say, $\beta = 0.05$), the ratio of the quantiles calculated from (4) and that of its corresponding exponential limit depends on $\alpha$ alone. When we increase $\alpha$, the Pareto quantiles grow, while the corresponding exponential quantiles stagnate. When we change $\beta$, both quantiles simultaneously change by different amounts, but they remain perfectly proportional to one another. Note too that the estimation of a particular, large quantile (return level) amounts to finding the intersection of a line and a function which it is (locally) nearly parallel to. Upon realizing this, it is clear why we should feel generally uncertain about the estimate of a large return level: a seemingly small change in $\alpha$ can shift this point of intersection quite drastically.

### Validation

To assess how well TED fits the data, we used a combination of statistical testing and scientific relevance, as suggested by the American Statistical Association^25^. In addition to analysis of p-values, we performed a comparison of empirical and TED-estimated 25-year return levels of Event total precipitation (Eq. 8) at each of the 4307 stations across the WUS.

#### Overall goodness-of-fit

According to a sequence of non-parametric goodness-of-fit tests, TED fits the Events recorded at at 87% of stations overall, and at 91% of those stations where we expect the model to fit: namely, those which exhibited good fit in the marginal distribution for duration and for which the univariate Pareto II distribution fit the Exceedeances which composed Events. Considering Figs. S1 and S2 together, we see broad spatial agreement on the decisions about the goodness-of-fit that this implies. A notable exception is apparent over much of Oregon and Washington where TED falls short of the expectations set by the fit of relevant marginal distributions. The spatial coherence of these hypothesis rejections suggests that there is an issue with how we have applied TED over some of Oregon and Washington. The details of the hypothesis testing which informed our overall goodness-of-fit assessment are covered in the supplemental materials.

#### Relative error of 25 year return periods

**Fig. 4:**
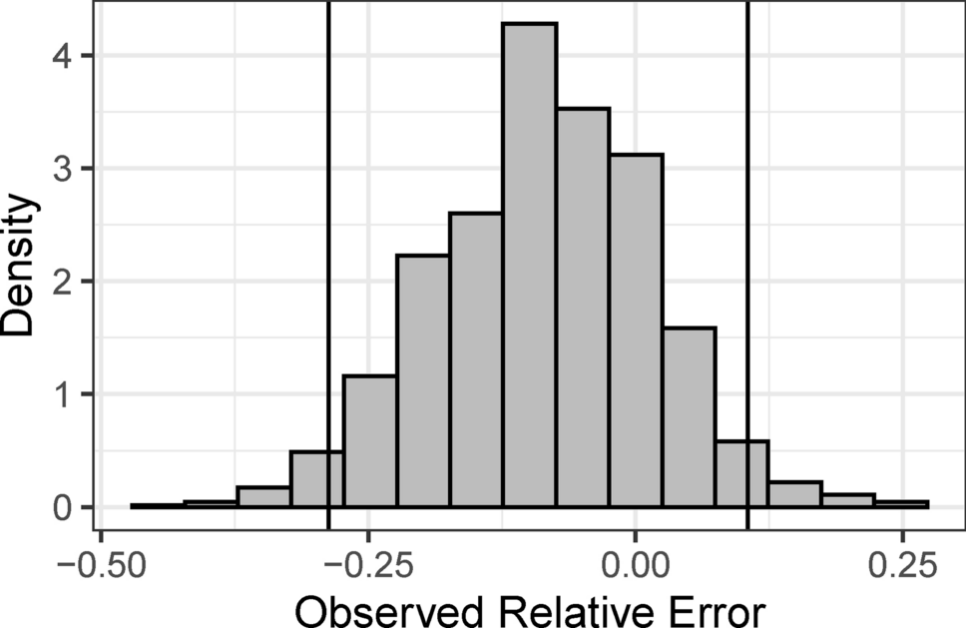
Histogram of the relative error of the theoretical 25 year return level of Event total precipitation vs. the corresponding observation; the calculated value is (theoretical-observed)/observed; considerations for this calculation for finite samples are detailed in “Interpreting comparisons of large theoertical and empirical quantiles” in “Methods” section; vertical lines denote the 2.5 and 97.5 percentiles of all observed relative errors.

To assess the practical fit of TED we computed relative error of 25 year return levels of Event totals. Figure 4 shows this relative error for 1366 (out of the total 2024) stations with at least 25 years of observations and for which the Event magnitude marginal fits the data ($p\ge 0.05$). The details of the relative error calculation are in “Interpreting comparisons of large theoertical and empirical quantiles” in “Methods” section. The relative error turned out skewed to the left as only 19% of errors are positive which indicates an underestimation bias of TED for Event totals. However, this underestimation is slight, as the median relative error is $-\,8.3\%$ and 95% of relative errors lie between $-\,29$ and 11%.

In addition, we computed 95% confidence intervals for the 25 year return periods using parametric bootstrap at every station. The endpoints of those intervals were taken as the 0.025 (low) and 0.975 (high) quantiles of the 25 year return level (parametric bootstrap) sampling distribution. We found that 97.3% of the observed 25 year return periods are covered by these intervals.

The analysis of relative error and its sampling distribution does not separate the uncertainty in the parameter estimates, which is difficult to estimate for technical mathematical reasons (see Kozubowski et al.^13^ for a mathematical discussion of this issue in a closely related problem).

### Application I: return periods of large event totals

**Fig. 5:**
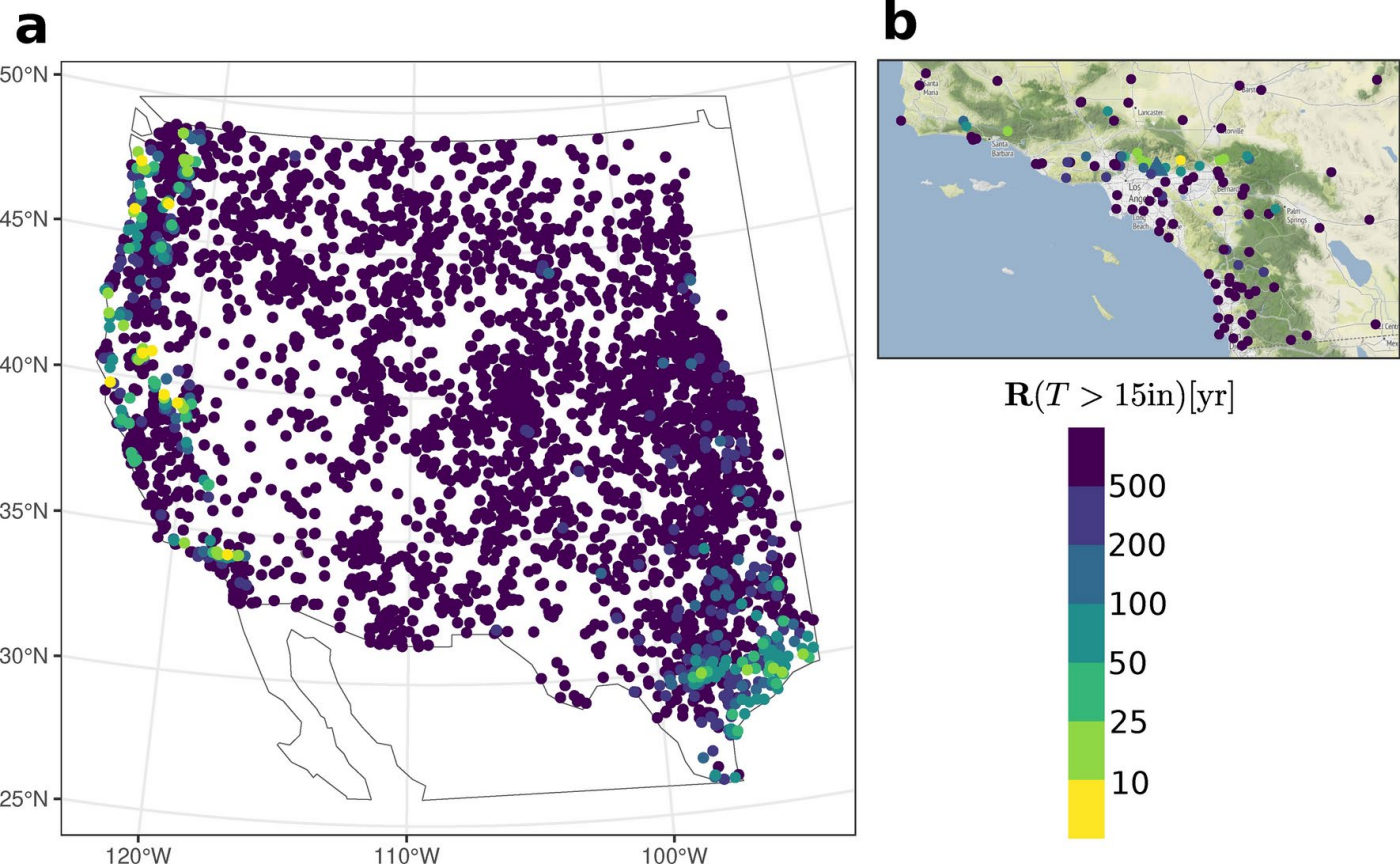
(**a**) Estimated return periods for exceeding a 15 inch (381 mm) total accumulation in a single Event; (**b**) inset focuses on southern California, with the point for San Gabriel Canyon Pumphouse distinguished from the others by its triangular shape and large size.

The TED-derived return periods for extreme Event total precipitation exceeding 381 mm (15 inches) have a coherent spatial distribution in Fig. 5. Such extremes are expected around the Gulf Coast of Texas with an expected frequency of 10–50 years due to hurricane landfalls on that flat low-lying flood-prone coastline (although the supplemental section Season-specific thresholds and parameters and Supplemental Fig. S7a,b suggest these Events totals can occur slightly more frequent than once every 10 years). The west coast, however, is impacted by more than its share of such inordinate extremes, where they are expected to occur with return periods of under 10 years at some mountainous locations and 25–50 years along the Coastal Ranges and the region’s major mountain chains—Cascade Range of Washington and Oregon, as well as the Sierra Nevada of Northern California and Transverse Ranges of Southern California. All these topographic features are known to promote heavy and extreme orographic precipitation due to ARs^21,23^, which produce the most extreme precipitation and the vast majority of flood damages along the North American west coast^26^. Figure 5b, focusing on the Transverse (east-west-oriented) Ranges of Southern California, highlights this tethering of extreme precipitation events to topography in that the 15 inch Events occur most frequently on the windward slopes of prominent topography. In contrast, low-lying coastal, valley, and leeward or less prominent mountain locations rarely record such precipitation totals. The TED is clearly sensitive to the strong topographic dependence of the largest regional precipitation extremes, which suggests that interpolation of TED parameters to ungauged locations should be possible as long as storm type, preferred atmospheric river orientation, and topography are appropriately taken into account.

### Application II: a comparison of historic rainfall events in the San Gabriel Mountains

There has been a pluviometer at the San Gabriel Canyon Pumphouse on the northern end of the San Gabriel Valley since 1916 (and possibly earlier). It is recorded to have been at the same spot from 1931 to 1999, when it was moved 67 m. Throughout, the reported elevation remained constant^27^. From this station, we have 103 water years in which 3 or fewer days of data are missing from 1 October through 30 April, when most of the precipitation falls.

The observations clearly illustrate the year-to-year variability in annual total precipitation. The mean water-year total is 546 mm (21.5 in), but the average deviation from this in any given year is 262 mm (10.3 in). This is because precipitation accumulated during an individual Event can almost reach the climatological value of total precipitation for an entire water year, which is typical of Southern California and the Southwest in general (see Dettinger et al.^21^ Fig. 2). Table 1 shows the trivariate summaries and totals of four historical Events: those with the top three recorded totals as well as a comparatively mild recent Event.

**Table 1:** Trivariate descriptions of four historic rainfall Events as they were observed at the San Gabriel Canyon PH pluviometer; the columns for X and Y have the event magnitude and maxima in mm, with the actual precipitation totals and maximum rates (that is, with sub-threshold precipitation added back in) in parentheses; the column for N shows Event duration in days.

Event	X [mm]	Y [mm]	N [days]
27 Feb–3 Mar 1938	293 (377)	148 (164)	5
13–19 Feb 1980	370 (487)	97 (113)	7
8–11 Jan 2005	315 (382)	113 (130)	4
4–7 Feb 2024	202 (270)	130 (146)	4

In March 1938, the San Gabriel Valley was at the center of a catastrophic flood affecting much of Southern California, including Los Angeles, San Bernardino, Riverside, and Orange Counties. Despite previous investments in flood control, the Los Angeles and Santa Ana Rivers overtopped their banks and eroded their existing channels. In Los Angeles, 1/3 of the land area was flooded, 6000 homes were destroyed, and at least 115 people died^28^. In Orange County, most of the bridges along the Santa Ana River were destroyed, Anaheim was flooded, and an untold number of people living along the river were killed^29^. After this flood, the LA River was channelized to its current concrete-lined form and previously agreed-upon flood control plans in Orange and Riverside counties were greatly enhanced^28,30,31^. The precipitation Event which caused this flooding is the main feature in Fig. 1.

From 13 to 20 February 1980, a rapid sequence of six cyclones brought with it a persistent precipitation Event and widespread flooding across Southern California and Arizona^32^. At San Gabriel Canyon PH, the largest Event total among all Events was recorded. Notably, the authors of a detailed meteorological and hydrological case study^32^ stated that “the February 1980 floods in southern California and central Arizona were caused by the cumulative effect of precipitation events, each of moderate and occasionally high intensity, and not by extreme rainfall of short duration. Examination of NOAA hourly precipitation data further revealed a lack of extreme events with durations of 1 to 24 hours, a characteristic that has been recognized as associated with winter precipitation brought about by extratropical cyclones”.

In January 2005 an Event with a total very similar to the 1938 Event proved to be a lot less damaging, though accounts from the time note deadly landslides^33^, power outages, and major rail shipping disruptions^34^. It was considered an official catastrophe by insurance companies and a state of emergency was declared in Southern California^35^, but the damage proved to be more localized and short-lived compared to 1938 overall^33^, probably in no small part due to the flood control infrastructure built in the wake of 1938.

Most recently, in early February 2024, an Event lasting 4 days garnered much attention due to large daily through 3-day accumulations, including the second-ranking three day total in Downtown Los Angeles^36^. At San Gabriel Canyon PH, there have been four shorter (3 days or less) Events with larger totals than that of 4–6 February, and there are other heavy 3-day accumulations embedded within longer Events, as well. This Event was less destructive than the others we have described^37^ and does not stand out probabilistically, as we learned by applying the TED. Tables 2 and 3 highlight how the various characteristics defining Events can combine to make Events unusual.

**Table 2:** Return intervals (in years) of the maximum daily precipitation accumulations recorded in each event with probabilities calculated four different ways: (i) Empirical CDF of Event Maxima, (ii) Univariate Pareto II CDF with parameters from MLE fit to all Exceedances, (iii) Univariate Pareto II CDF with parameters from MLE fit only to Event maxima (a crude way of “declustering” dependent Exceedances), (iv) TED Marginal Y, MLE fit; intervals show empirical quantiles 0.025 and 0.975 from resampling Events and Exceedances 1000 times with replacement and re-estimating parameters; sample sizes are 739 and 1066 for Events and Excedances, respectively.

Event	$\textbf{R}\big (\frac{1}{N}\sum \mathbb {I}{\{y_i>y\}}\big )$	POT1		POT2		$\textbf{R}(Y>y)$	
1938	22	25	(16, 63)	28	(16, 70)	23	(15, 44)
1980	6	5	(4, 7)	6	(5, 9)	5	(4, 8)
2005	10	9	(6, 15)	10	(7, 17)	9	(7, 13)
2024	11	15	(10, 29)	17	(11, 34)	14	(10, 24)

Table 2 shows return periods for the largest daily accumulations occurring during the Events. Several different methods are basically in agreement with one another as well as with the (roughly) analogous estimates from NOAA Atlas 14, wherein the peak day of the 1938 Event has a return period of 25 years and that of the 2005 Event is 5–10 years. Now, it is worth noting that there *is not* a theoretical correspondence between all of the return interval estimates. “POT1” comes from a Univariate Pareto II fit to all Exceedances, while “POT2” is from the same distribution fit to different values: the largest daily total in each Event. This is a very crude form of “declustering” in a peaks-over-threshold analysis^1^. We are proposing a different distribution for these same values: the Y marginal of the TED. We included these estimates side-by-side, along with an empirical estimate, to show that applying the similar methods to different data (POT1 vs. POT2 vs. NOAA14) or different methods to the same data (POT2 vs TED) provide broadly consistent results. Overall, not all of the Events we are scrutinizing had remarkably high maximum intensities, but at least three of the four were remarkable in other ways, as we shall see.

**Table 3:** Various return periods and a probability calculated under TED; return interval values $\textbf{R}(\dots )$ are in years; Bootstrapped 0.025 and 0.975 quantiles are parenthesized.

Event	$\textbf{R}(X>x)$	$\textbf{R}(N\ge n)$		$\textbf{R}(X>x, Y>y)$	$\textbf{R}(T>t)$		$\textbf{P}(Y>y\mid N= n)$ (%)	
1938	134	16	(10, 29)	178	173	(86, 525)	2.2	(1.2, 3.3)
1980	429	135	(68, 360)	434	770	(293, 3700)	12	(9.7, 15)
2005	190	6	(4, 8)	204	186	(92, 580)	4.7	(3.2, 6.2)
2024	28	6	(4, 8)	43	33	(20, 68)	2.9	(1.7, 4.1)

Table 3 shows return periods for further Event types we might calculate with a TED. As is apparent in Table 1, the magnitude X of the 1938 Event is much less than that of the 2005 Event, but their actual total accumulated precipitation amounts are very similar. All aspects of trivariate Events can likewise be distinguished and their relative peculiarity judged with a single distribution of four parameters. By our count, there are at least hundreds distinct and meaningful probability statements for which a simple application of the TED CDF can calculate a value. This abundance of potential proabability statements and return periods is just one reason why return periods are a troubled concept. Overall, the TED, its marginal, and conditional distributions let us get to the heart of what makes each Event special: for example, 1938 for its high maximum intensity, even for an even for an Event of its duration, 1980 for its mild maximum intensity, despite its very large magnitude and duration, and 2005 due to its high magnitude, given its more modest duration ($\textbf{P}(Y>y_{1938}\vert N=n_{1938}) = 2.2\%$, $\textbf{P}(Y < y_{1980}\vert X>x_{1980},N=n_{1980})=3.5\%$, $\textbf{P}(X>x_{2005}\vert N=n_{2005})=0.77\%$).

## Conclusion

### Current applicability

TED is applicable to one-day-and-longer precipitation Events. We have demonstrated its applicability in the Western US—a well-sampled mountainous region. In a Mediterranean climate region, the basic approach we have presented is sufficient, but in areas with multiple rainy seasons, we currently recommend seasonal binning of precipitation observations before estimating thresholds and parameters. All results presented in the main text were based on the simplest possible application of the model with a threshold and parameter estimate which does not vary with the time of year. See the Supplemental section “Season-specific thresholds and parameter estimates” for an example of accounting for seasonality.

TED allows us to model whole physical Events, rather than temporally isolated data points or aggregations over prescribed, arbitrary durations. It allows us to assess the probabilities, return levels, and return periods of the most relevant constituents of an Event—duration, maximum intensity, and overall total magnitude—independently, simultaneously, or conditional on each other.

### Broader applications

The TED we have put forward is quite general. Randomly stopped sums and maxima of Exceedances can in principle be applied to just about any random variable for which extended excursions over a threshold are of interest (e.g. heatwaves or floods). Daily precipitation totals are merely the first variable we decided to look at, due to the abundance of data over a wide region, the authors’ familiarity with it, and also in light of the results of Dettinger et al.^21^, who suggested that understanding a few multi-day precipitation episodes per year would go a long way toward understanding hydroclimate variability and precipitation risks in California.

### Next steps for TED and its applications to precipitation

We have presented the application of the TED in its most “raw” form. Mutual independence between (though not within) Events was assumed for the estimation of the parameters via the maximum likelihood method. The parameter values were assumed to not vary with the season or any other climate-relevant factors. Despite this simplicity, the TED is widely applicable, as it is representative of the process which generated the data. Random vectors of random lengths, with dependence on their environment and between their constituent elements, with a tendency to produce very large values relative to their mean are summarized in terms of their length, sum, and maximum. The parameters of the TED all have clear interpretations, as $\beta$ is closely related to the mean, $\alpha$ simultaneously describes a propensity for “outliers” (univariate interpretation, Eq. 4) as well as a “shared environment” affecting constituents of an Event (vector interpretation, Eq. 2), and *p* and *q* characterize duration. How can we use and improve this model to better understand precipitation within a varying and changing hydroclimate?

A statistical model of the parameters in terms of covariates (a vectorized generalized linear model and/or more domain-specific spatial statistical models) would allow us to use information from places with long records in other locations with shorter or no records. Likewise, the parameters could be interpolated on a grid. Statistical modeling of parameters has other benefits, including the provision of an objective characterization and tempering of the undue influence of the presence or absence of large observations (e.g. the presence of the February 1980 Event at a station with a very short record) on the estimation of $\alpha$.

Thus far, we have applied TED in a stationary manner. However, since climate varies on multiple timescales due to natural variability and is changing due to human influence, it is non-stationary. In particular, in the western US, precipitation—including heavy and extreme events—varies strongly on interannual and decadal timescales due to El Niño Southern Oscillation^38^ and the Pacific Decadal Oscillation^39,40^. We also expect the hydroclimate of the region to change with future warming in two fundamental ways—with precipitation becoming generally less frequent but intense precipitation specifically from atmospheric rivers becoming more extreme^41^, though this has not yet been observed^42^. The flexibility afforded by TED, particularly our ability to include covariates representing the climate state and proportional mixtures of storm types, can further improve the fit of the TED to make it applicable for studying the role of Events in a varying and changing climate. That would constitute important advances in seasonal climate prediction and climate change projection. Explicitly accounting for non-stationarity and using spatial dependence to interpolate TED parameters to ungauged locations will be the goals of our future efforts.

In the meantime, however, the simpler application of TED presented here can be used to assess probabilities and return periods of precipitation Events—whether observed or precisely specified for specific applications—at locations with adequate data and nearby locations with similar climate and topography. The trivariate characterization allows us to describe different flavors of precipitation Events with respect to their duration, maximum intensity, and magnitude, and the TED objectively assigns probabilities to specific Events, allowing us to quantify exactly how unusual they are.

## Methods

### Probability distributions

#### Vectors of exceedances: multivariate pareto type II

Our fundamental model of the daily precipitation Exceedances $\textbf{E} = ( X_1, \ldots , X_n)$ that make up an Event of length *n* is *multivariate Lomax* or *multivariate Pareto Type II* (MVPII) (see, e.g. Arnold^43^, Hutchinson^44^, Nayak^45^, and Yeh^46^, and references therein). Conditionally on $N=n$ (for a given duration), the (conditional) n-variate distribution of $\textbf{E}\vert N=n$ is given by the joint PDF:

1 $$\begin{aligned} f_{n}(x_1, \ldots , x_n;\;\alpha , \beta ) = \frac{(\alpha \beta )^n \Gamma \left( \frac{1}{\alpha } + n\right) }{\Gamma \left( \frac{1}{\alpha }\right) } \left( 1 + \alpha \beta \sum _{i = 1}^n x_i \right) ^{-\frac{1}{\alpha } - n}, \,\,\, x_i \in \mathbb R_+, \,\,\, i=1, \ldots , n, \end{aligned}$$

where $\beta >0$ is a scale parameter and $\alpha \ge 0$ is a tail (or shape) parameter. In the limiting case $\alpha = 0$, the components of $\textbf{E}$ are understood as independent and identically distributed (IID) exponential with scale (reciprocal mean) $\beta$.

To understand $\alpha$ and the special limiting case of $\alpha \rightarrow 0$, the following stochastic representation^47^ is useful:

2 $$\begin{aligned} \textbf{E} = (X_1, \ldots ,X_n) {\mathop {=}\limits ^{d}} \left( \frac{E_1}{Z}, \ldots , \frac{E_n}{Z} \right) , \end{aligned}$$

where $\textbf{E}$ is multivariate Lomax with PDF (1) and the $\{E_i\}$ are IID exponential variables with mean $\beta ^{-1}$. The quantity *Z* is a “mixing” gamma random variable, independent of the $\{E_i\}$, with both shape and scale parameters equal to $\alpha ^{-1}$ and the PDF given by:

3 $$\begin{aligned} g(x;\;\alpha ) = \frac{(1/\alpha )^{1/\alpha }}{\Gamma (1/\alpha )} x^{\frac{1}{\alpha } - 1} e^{-\frac{1}{\alpha } x}, \ \ \ x\in \mathbb R_+. \end{aligned}$$

In the limiting case $\alpha =0$, the variable *Z* in (3) equals to 1 (a degenerate constant), and $\textbf{E}$ contains *n* IID exponentially distributed components $E_1, \ldots , E_n$.

When the MVPII model appeared in the context of reliability theory, the variable *Z* described the effect of a common environment on structurally-independent, individual components of a system with individual lifetimes represented by the $\{E_i\}$^45,48^. A similar interpretation, with 1/*Z* representing a common (multiplicative) *background risk*, has made this an attractive model in finance and insurance^49–53^. In terms of precipitation, we may interpret *Z* as representing an atmospheric circulation mode (e.g. Pacific North American pattern^54,55^) or perhaps a more slowly-varying climate state (e.g. ENSO). Which of these interpretations is more useful for precipitation will become more apparent when we create a model for the parameters. In modeling work, the mixing variable interpretation of $\alpha$ and *Z* should grant practitioners flexibility in accounting for the distinctions between Events and the seasons they are embedded in as well as for the dependence of observations within Events.

There is yet another consequential interpretation of $\alpha$. The one-dimensional (marginal) distributions the $\{X_i\}$ are *univariate* Pareto Type II^43^, given by the survival function (SF)

4 $$\begin{aligned} \textbf{P}(X_i>x) = \left( 1 + \alpha \beta x\right) ^{-\frac{1}{\alpha }}, \,\,\, x\in \mathbb R_+, \end{aligned}$$

which arises in POT theory^1,3,4^, as the asymptotic distribution of (unbounded) Exceedances. In this context, the random variables $\{X_i\}$ in (4) are said to have a power law or “heavy” tail with tail parameter $\alpha$. The larger the $\alpha$, the heavier the tail, which is another way of saying that the larger the $\alpha$, the larger the probability that $X_i$ exceeds any given value *x*. The influence of $\alpha$ is immediately clear in Fig. 3b,c, wherein forcibly setting $\alpha = 0$ (i.e., assuming an exponential distribution in a POT analysis of daily precipitation Exceedances) would lead us to believe that the record largest precipitation observation is about 1/4 as common as we believe it is when we more sensibly estimate $\alpha$ using observations. Additionally, it is important to note that, in contrast with the model developed by Furrer et al.^5^, where the GPD was applied only to the first value within an extreme event, our approach applies the GPD to all values within the event, consistent with the POT Theory.

#### Event durations: hurdle geometric

The discrete distribution we use for Event durations is called “hurdle geometric” and is given by the probability mass function:

5 $$\begin{aligned} g(n) = \textbf{P}(N=n) ={\left\{ \begin{array}{ll} q & n=1\\ (1-q)p(1-p)^{n-2} & n\in \{2,3,\dots \}. \end{array}\right. } \end{aligned}$$

This distribution arises naturally if we play a game with two biased coins. $C_q$ and $C_p$ have probabilities *q* and *p* of landing on heads, respectively. First, flip $C_q$. If it shows heads, the game is over in one turn, i.e. Event duration is 1. If it yields tails, flip $C_p$ until you see heads. If all the flips were independent, the probability that the game lasted precisely *n* turns is given by equation (5). A contrived coin-flipping game is certainly not the only process which can give rise to such a distribution: indeed, this distribution has already been successfully applied to episodes of growth of an index stock prices^18^, arises approximately in durations of excursions over thresholds in stationary time series^56^, and it apparently also provides a good description of precipitation Event durations (see Supplemental Material; Goodness-of-fit; Model appropriateness). It should be noted that, compared to the geometric distribution, which is commonly applied to event duration in similar contexts (Raha and Ghosh^56^, Furrer et al.^5^, Keellings and Waylen^57^, Mondal and Mujumdar^58^), the Hurdle Geometric model offers more flexibility in accounting for events with durations of $N=1$, which may occur with frequencies inconsistent with the standard geometric distribution. At the same time, the geometric distribution is included here as a special case when $p=q$.

#### Events summaries: the TED random vector

The vector of magnitude *X* and maximum *Y* of an Event is mathematically a *randomly stopped* sum and maximum, since it is composed of conditional distribution of $(X,Y)|N=n$ and a discrete distribution of the Event length *N*, which is independent of the components of $\textbf{E}$. Indeed, we have:

6 $$\begin{aligned} {\textbf {T}}=(X,Y,N){\mathop {=}\limits ^{d}} \left( \sum _{k=1}^{N} X_k, \bigvee _{i=k}^N X_k, N \right) , \end{aligned}$$

where, in this work on daily precipitation, we assume the $\{X_i\}$ to have an MVPII distribution. Sums and maxima of this kind were comprehensively characterized mathematically by Arendarczyk et al.^15^. In particular, the description included the PDF, CDF, marginal and conditional distributions (e.g. $(X|Y = y)$, $(X, N) | Y = y$, $(Y, N) | X = x$, $(X, N) | Y = y$, $X | Y=y, N=n$, $Y | X=x, N=n$, etc. ), moments, covariance matrix, maximum likelihood estimators, and other properties. Note, that when $n=1$, we get a degenerate distribution on the line $x=y$, and for $n \ge 2$ we obtain a proper trivariate distribution for (6). Note also that, due to the heavy tails, the mean of *X* and *Y* exists whenever $0\le \alpha <1$, and their covariance matrix exists for $0\le \alpha < 1/2$. These theoretical limitations on $\alpha$ have *not* been a hindrance to the study of daily precipitation Exceedances (see Fig. 2). Note, that although we model *N* with a hurdle geometric distribution, in general one can use *any* discrete distribution on positive integers for *N*. Further, the distribution of ${\textbf {T}}$ can always be described as a mixture of a degenerate distribution when $N=1$ and a proper trivariate distribution for $N \ge 2$ (see Remark 4.2 in Arendarczyk et al. (2018)), which makes our hurdle approach quite natural.

Given our representation (6) of Event summary vectors, the fact that the distribution of observed daily precipitation Exceedances is indeed univariate Pareto II (4)^12,14^, and that Event durations follow (5), it is natural to suppose that the joint distribution of $\textbf{T}$ would be given through conditioning as:

7 $$\begin{aligned} F(x,y,n)=\textbf{P}(X\le x,\,Y\le y,\,N\le n) = \sum _{k=1}^{n}\textbf{P}(X\le x,\,Y\le y\mid N=k)\textbf{P}(N=k)=\sum _{k=1}^{n}F_k(x,y)g(k), \end{aligned}$$

where $F_k(x,y)$ is the joint CDF of the sum and maximum of a Pareto II vector of length *k* given by Arendarczyk et al.^15^ and *g*(*k*) is the hurdle geometric mass function in (5). The distribution with CDF (7) may be called Hurdle-geometric Sums & Maxima of Pareto variables (HGSMP). It is an example of a TED, and throughout this work we merely refer to it as “TED”.

Note that if summarized Events ${\{\textbf{T}_i\}}$ are mutually independent and obey (7), then (5) is the distribution for Event durations and the Exceedances (specifically, those occurring in distinct Events) are IID following Pareto II. The converse is not true nor are we claiming it is so. However, it inspires our thinking in that the proposed TED is perhaps the most straightforward model for Event summaries which is consistent with observed Event durations and previous POT work on precipitation Exceedances. Furthermore, ill fit of either the purported Event duration distribution or Exceedance distribution provide some insight as to *why* the TED may not be directly applicable at some locations or under an overly-simplistic (in this case, constant) model of the parameters.

#### Coherence of TED-modeled events

A TED is built from two basic ingredients: (i) the marginal distribution of *N* and (ii) the conditional distribution of *X* and *Y* given that $N=n$. The joint probability density function (PDF) of *X*, *Y*, and *N* is then obtained in a standard way by multiplication of those corresponding to (i) and (ii) (Eq. 7). However, it is important to note that this joint distribution governs all marginal and conditional distributions, and in particular the conditional distribution of *N* given the values of *X* and *Y*. In other words, one can make inference on the duration *N* given observed values of $X=x$ and $Y=y$

Thus, while it is true that we selected a model for the duration first when building the trivariate distribution, ultimately there is correlation between all parts of the model. That is, there is association between *X* and *N*, *Y* and *N*, and *X* and *Y*. This correlation and the structure of the model itself ensures that the model is coherent so that when the values of $X=x$ and $Y=y$ are close, a small *N* is more likely (and the strength of this relationship depends on $\alpha$). An advantage of our model is precisely that internal coherence and association between its components, together with random *N* reflecting the actual duration of a storm or any precipitation Event above the selected threshold.

#### Event total accumulations: a TED-derived distribution

The magnitude of an Event is the sum of consecutive Exceedances. This is different from the total precipitation accumulated during an Event. Given a threshold and a duration, it is easy to reconstitute the actual total by adding the below-threshold part back in. It is also not difficult to calculate exceedance probabilities of Event totals based on TED, leading to:

8 $$\begin{aligned} \textbf{P}(T>t) = \sum _{k=1}^{\infty }\textbf{P}\big (X + k\mu >t\vert N=k)\textbf{P}(N=k). \end{aligned}$$

### Threshold selection for defining events

Note that any sufficiently high threshold may be chosen, but it is difficult to know exactly how high a threshold is high enough. Threshold selection for POT and for the present type of Event analysis is not identical. In the POT threshold selection step, there is a bias-variance trade-off wherein raising the threshold decreases our sample size and the variance in parameter estimates. This makes theoretical results about parameters estimates under increasing threshold difficult, but not impossible, to interpret. For Events, there is another issue in which large thresholds unduly fracture Events. For extremely high thresholds, there are only likely to be Events of duration 1, which defeats the purpose of a multivariate analysis in the first place.

Most of the “bulk” analysis and estimation was also conducted on the percentiles 50, 60, 80, 85, 90, and 95 and informal checks^1^ on the constancy of parameter estimates (with respect to threshold) as well as their spatial patterns were conducted.

Ultimately, we settled on the 75th percentile. This is due partly to precedent of earlier work^12^, the fact that we were satisfied with the behaviour of the parameter estimates when the threshold varied, and that visual verification of QQ plots and GoF metrics of a univariate Pareto II distribution for Exceedances confirmed that the threshold was sufficient.

### Parameter estimation

There are many ways one could estimate parameters of a hypothesized distribution given observations. We opted for classic maximum likelihood estimation (MLE) and, for the ease of estimation, assumed that Events are all mutually independent (*although do note that Exceedances within the same Event are not assumed to be independent*). Due to our simplifying assumptions, the form of the CDF (7), and previous work^15,18,59^, we were able to estimate the parameters of the duration *N* and those describing the sums and maxima $(X,Y)\vert N=n$ separately.

$\hat{p}$ and $\hat{q}$ have intuitive, closed-form solutions^18^:

9 $$\begin{aligned} \hat{q} = \frac{N_1}{N} \text { and } \hat{p} = \Bigg [\frac{1}{N_{>1}}\sum _{i=1}^{N}(n_i-1)\mathbb {I}_{\{n_i>1\}}\Bigg ]^{-1}, \end{aligned}$$

where *N* is the number of Events, $N_1$ is the number of Events of duration 1 time increment, $N_{>1}$ is the number of Events longer than 1, and $n_i$ is the duration of Event *i*. The estimated value $\hat{q}$ is natural, given definition of *q*: the probability that an Event last only one day. $\hat{p}$ is merely the MLE of the parameter of a geometric distribution, with adaptation to the fact that this is a “hurdle” geometric distribution, which offsets our counts by 1.

$\hat{\alpha }$ and $\hat{\beta }$ are more complicated^15,59^. Although the MLEs always exist, they must be found numerically, and these estimates are not known theoretically to be unique. The result of a preliminary step in the maximization procedure indicates whether or not the limiting case of $\hat{\alpha } = 0$ would maximize the likelihood function. In this case, $\hat{\beta }$ is simply $\overline{N}/\overline{X}$, which is intuitively consistent with a scale parameter of the IID exponential variables which constitute Events when $\alpha = 0$. In the Pareto case ($\alpha >0$), $\hat{\alpha }$ and $\beta$ are found through numerical optimization. An interesting property of this parameter estimation approach (which is shared with the exponential case) is that the maxima of Events $\{Y_i\}$ do not make an appearance in any step of the procedure: only the Event durations and magnitudes are considered when estimating parameters which also pertain to the maxima.

### (Non)stationarity of precipitation

Precipitation varies seasonally, interannually, and on longer timescales, both in its frequency of occurrence and its character. The presentation of the sensible structure of the TED and its broad applicability to daily precipitation are the main purpose of this work, and general (not conditioned on covariates) Event-level probabilities are the quantities we present. The inclusion of covariates, e.g. to represent seasonality, over such a large and hydroclimatically diverse region is not our present goal and would distract from our presentation of the TED.

Nonetheless, it is worth commenting on the potential issues stemming from how we have temporarily ignored seasonality by using constant thresholds and parameter estimates. To begin with, we *do not* claim that precipitation is stationary within or between years. We do, however, believe that the TED we present, given its inclusion of the tail parameter $\alpha$, is well equipped to implicitly mix probability distributions of precipitation across seasons. Cavanaugh et al.^14^ showed that this is the case for the Pareto II distribution applied to daily precipitation Exceedances. In a basic POT analysis, one can calculate valid 100 year return levels without any reference to the varying probability within years with which the 100 year return level is exceeded. An explicit treatment of seasonality is not necessary for the estimation of “bulk” probabilities.

Some remaining concerns are that by defining a single threshold and using a single parameter estimate, we are ignoring some types of Events (those which fall below a constant threshold) and conflating distinct types of Events which occur at different times of year. The former amounts to the assignment of conditional probabilities to different types of storms at different times of year, which is not our immediate goal. However, the latter is immediately relevant. The conflation of different event types in different seasons amounts to estimating a single set of parameters for what may be better represented as a mixture of two or more distributions. The concern is that this may bias the parameter estimates and probabilities. We note that is no more an issue with TED than it is with any statistical analysis which neglects a potentially-important covariate.

The Supplemental section “Season-specific thresholds and parameters” shows examples of seasonal binning of the data, followed by the application of TED. The is that in a Mediterranean climate, such as in California, both a constant threshold and parameter estimate are advisable for most applications we can imagine. Elsewhere, in regions of more complex seasonality, it may be worth binning the data by season.

### Interpreting comparisons of large theoretical and empirical quantiles

Let *A* be a statement about an Event, such as “maximum intensity is greater than 30 mm/day”. We have been supposing Events are IID, so a single number $p = \textbf{P}(A)$ suffices as the probability that this statement about any Event is true. In turn, $p^{-1}$ is the expected number of Events which occur until *A* is true. If there are *m* Events per year (on average), it takes an average of $(pm)^{-1}$ years for *A* to be true. This means that given a desired return interval *i* and Event occurrence rate *m*, we can calculate a target probability *p* as $(im)^{-1}$.

Our “observed” *i* year return level is then $\{x\}_{\lceil np\rceil }$, where $\lceil \;\rceil$ is the ceiling or “round up” operation and *n* is the total number of Events observed. This definition allows us to think of observed return levels directly in terms of order statistics, which is especially helpful whenever we wish to account for the fact that our sample sizes are limited. For instance, given a sample of size 100, it would not make sense to *directly* compare the 99th largest observation of this sample to the 0.99 quantile of the distribution which we believe to have generated the sample. While, the 99th largest observation of this sample, $\{x\}_{\lceil 99\rceil }$, would be a natural and intuitive estimate of the 99th percentile, we are not equally likely to underestimate it as we are to overestimate it. In the IID setup for any distribution *F*, $$\textbf{P}\big (\{x\}_{\lceil np\rceil } < F^{-1}(p)\big )=\sum _{\lceil np \rceil }^n \left( {\begin{array}{c}n\\ k\end{array}}\right) p^k(1-p)^{n-k}$$, which is about 0.74 when $n=100$ and $p = 0.99$. Furthermore, the distribution of the magnitudes of the differences between empirical quantiles and theoretical quantiles depends on the underlying theoretical distribution which we believe to have generated the sample. In the case of heavy-tailed distributions, large p, and small n (largeness of p is relative to n and vice versa), we need to be extra careful, as order statistics of samples can be quite noisy. With all of this in mind, all observed return levels are compared to the median of the corresponding order statistic distribution, given by:

10 $$\begin{aligned} G_{n,p}(x)=\mathbb {P}\big (\{X\}_{\lceil np\rceil } \le x\big ) = \sum _{k=\lceil np\rceil }^{n}\left( {\begin{array}{c}n\\ k\end{array}}\right) F(x)^k(1-F(x))^{n-k}, \end{aligned}$$

where *F* is the CDF of the assumed underlying distribution.

Any quantile of $G_{n,p}$ may be calculated numerically and this is precisely what we did in “Relative error of 25 year return periods” in “Results and discussion” section. The “theoretical” values we compared $\{x\}_{\lceil np\rceil }$ to were the 0.025 (low), 0.5 (median), and 0.975 (high) quantiles of $G_{n,p}$. “Relative Error” is the median minus the observed value divided by the median, and ${x}_{\lceil np\rceil }$s were considered “reasonable” whenever they landed between the low and high values.

## Supplementary Information

Supplementary Information.

## Data Availability

The daily precipitation observations are from the Global Historical Climatological Network Daily (GHCNd) dataset, which is available for free at https://www.ncei.noaa.gov/products/land-based-station/global-historical-climatology-network-daily.

## Declarations

### Companion R package

AW has written an R package, called “events”, which allows users to identify Events from timeseries and to analyze their distributions. The package is available on https://github.com/aweyant/events.git.
