# Supplementary material for: A holistic stochastic model for precipitation events
Source: Sci Rep. 2025 Feb 7;15:4595. doi: 10.1038/s41598-024-77031-3 (PMC11806066; doi:10.1038/s41598-024-77031-3)
Supplement: Supplementary file 1 — Supplementary Information. [file 41598_2024_77031_MOESM1_ESM.pdf]

Supplemental Material: A Holistic Stochastic Model for Precipitation Events

Alexander Weyant[1,*], Alexander Gershunov[1], Anna K. Panorska[2], Tomasz J. Kozubowski[2], and Julie Kalansky[1]

[1]Scripps Institution of Oceanography, (Department of) Climate, Atmospheric Sciences, and Physical Oceanography, La Jolla, California, 92037, United States
[2]University of Nevada, Department of Mathematics and Statistics, Reno, Nevada, 89557, United States
[*]aweyant@ucsd.edu

Validation

The theoretical and scientific case for the TED itself is strong (see **Introduction** and **Methods**). This notwithstanding, in order to assess the overall fit of the model to the data, we used a mixture of testing hypotheses and the assessment of the relative error of the calculated 25 year return periods. This mixed approach is suggested by the American Statistical Association[1], which recommends that scientific decisions should not be based solely on the p-values. Rather, all relevant scientific information and reasoning should inform decisions.

Hypothesis Testing

We performed hypotheses tests in order to assess the statistical fit of the model and, more importantly, to learn where the model, as currently applied, does not fit and the data requires a modified approach. Throughout this section, the goodness-of-fit tests are based on Dowd's Two Sample test[2], which compares two empirical (univariate) CDFs. The null hypothesis is that two samples arose from the same CDF. Thus, we compare observations to a simulated (synthetic) sample from the hypothesized distribution. The size of the synthetic sample is set to be ten times that of our observations. The sampling distribution of Dowd's statistic was approximated with bootstrapping with 2000 replicates (the default setting in Dowd's R package[3]). For our purposes, "good fit" means that the p-value from this test (or multiple, Bonferroni-corrected p-values, in the case of simultaneous tests) is greater than 0.05.

Setting Expectations or Model Appropriateness

A multivariate CDF fully describes all marginal distributions (and more). We would not expect a TED to fit the data if any of its marginals do not. One basic question, which arose even before the application of this TED was whether we would *expect* it to fit the data. Two necessary conditions in particular are easy to test:

1. whether or not the hurdle geometric distribution for durations fits
2. if the univariate Pareto II distribution fits the Exceedances; This distribution arises theoretically[4] and has been validated empirically[5,6], but we needed to replicate this for Exceedances at all stations

We simultaneously tested the hypotheses, which yielded S1 as well as many station-level QQ plots (not shown). The QQ plots verified that the tests were exposing fit deficiencies as we expected. If the null hypotheses were all true and the tests valid and sufficiently powerful, we would expect the points of hypothesis rejections ("false positives") to be distributed across the map in rough association with sample size at the stations, and to constitute about 5% of the total points. Overall, we *expect* the proposed TED to fit almost anywhere in the domain, but note that varying sample sizes, Type I errors, Type II errors, and spatial patterns in the test results somewhat confound this. Also, at locations where the trivariate distribution does not fit after all, there are clear explanations and remedies.

Trivariate Goodness-of-Fit

To look at the trivariate fit, we first performed three simultaneous hypothesis tests on all marginals (duration, magnitude, and maximum intensity). Figure S2 shows TED, as applied, fits at 87% of stations overall and at 91% of stations where we expected it to. However, there may be issues in exactly how we applied the TED over parts of Oregon and Washington. Note that the hypothesis tests are, fundamentally, testing whether or not modeled TED CDFs are identical to empirical CDFs. Rejection of the null hypothesis suggests an issue somewhere in the long process which culminated in the modeled CDF rather than the choice of parametric family of the CDF itself. Here, the treatment of the data was rather simplistic and we believe more sophisticated treatment (e.g. seasonal or meteorologically-informed modeling of parameters with respect to covariates) in Oregon and Washington would correct these issues.

In addition to goodness-of-fit of the marginal distributions, we also considered the overall multivariate goodness-of-fit on a subset of 1/10 of the stations by applying the distribution free multivariate two sample test of Baringhaus and Franz (2004)[7]. To implement the Baringhaus test, we used the the R package cramer[8] with the default configuration of 1000 bootstrapping replicates. We compared the actual sample to a synthetic sample of equal size based on the maximum likelihood estimated parameter values. The results of testing multivariate fit were consistent with the results of the tests on the marginals, so our results for simultaneous results on the marginals in fact apply to the truly multivariate case.

Uncertainty Figures

We selected a subset of 1/10 of all of the stations with records longer than 10 years, created 100 bootstrapped replicates of each, and re-estimated the parameters. Figures S4,S5 give an idea of how uncertain the parameter estimates are and what their sampling distributions look like in practice.

Season-specific Thresholds and Parameters

In this work, we have used a constant threshold to define Events, and have estimated the distribution's parameters without explicit consideration of the seasonal cycle of precipitation. Especially in light of the fact that different locations have different seasonal cycles of precipitation, we may wonder if the "one size fits all" approach of a constant threshhold and parameter vector unduly affects our calculated probabilities. The interannual variability part of this is well beyond the scope of this work, but the intra-annual (seasonal) aspect can be crudely approached by means of binning observations into three month seasons. We inspected three stations with distinct hydroclimates in the Southwest and found that considering seasonal binning has a minimal effect in a Mediterranean climate (with just one rainy season) but is more consequential elsewhere, where precipitation is produced by different types of storms in different seasons, as expected. This however, does not detract from the main points of this work: namely, that a heavy-tailed TED is suitable for describing precipitation Events, and that the TED allows us to calculate probabilities of the occurrence of Events based on joint, conditional, or marginal considerations of their characteristics.

Site Descriptions

We selected three long-term daily pluviometers in locations with different seasonal characteristics. Some of their metadata are listed in Table 1.

Grand Junction, in the Northeast of the Colorado Plateau and on the western foot of the Rockies, has a relatively flat seasonal cycle of precipitation (Fig. S8b). All months of the year, save June, have similar mean total precipitation.

Broadly viewed, Santa Rita Experimental Range sits on the edge of the Sonoran Desert and the Chihuahuan Desert. More locally speaking, it is on the Northeast foot of the Santa Rita Mountains, an "island" in the Madrean Archipelago of prominent peaks rising thousands of feet over the surrounding desert and grasslands. There are two rainy seasons (Fig. S8a) which, in the aggregate, somewhat blend in to one another in September and October: the monsoon season, which peaks in July and August, and the winter, which peaks in December through February. There is a lull in October and November between these two seasons, but their exact start and end dates vary between years. April, May, and June are the distinctly dry season between the end of Winter and the onset of the next Monsoon.

San Gabriel (in Southern California), as described in the main text, has a Mediterranean climate: it receives almost all of its precipitation in a single rainy season (Fig. S8c). Averaging over all years, this is between October and April, and peaking in December through March, but there is little regularity of precipitation in any of these months.

With respect to the "one size fits all" approach to threshold determination and parameter estimation, we should a priori expect San Gabriel to be the least problematic case, followed by Grand Junction, then Santa Rita. Explicitly, this comes from the expectation that San Gabriel, with its single winter rainy season, can be well described by a single distribution, whereas Santa Rita (especially) may require a mixture of distributions to describe the various rainy seasons. This being said, the TED we are proposing is is well-suited to mixing seasons implicitly through the tail parameter α. Previous work[6] indicates that locations with many kinds of precipitation at different times of year and α being positive within at least one season tend to have a positive α when a single threshold and parameter vector is estimated on observations from all seasons. From the point of view of daily precipitation exceedances, this implicit mixing is empirically successful (Fig. S1) - a single threshold and estimated parameter vector is suitable for describing daily precipitation exceedances at almost all stations in the domain.

Parameter Estimates

After binning days into three-month-long seasons (DJF, MAM, JJA, SON), we calculated the season-specific 75th percentiles of above-trace precipitation, defined events thus, and estimated parameters by maximizing the likelihood function. We repeated this on 500 bootstrapped replicates, and the result of this is displayed in Fig S9 as a point density plot[9] of marginal sampling distributions.

GHCN ID	Name	Lat	Lon	Elev. [m]	Period<br>Yrs Valid	Water Yr. Prcp<br>Mean (SD) [mm]
USC00027593	SANTA RITA EXPERIMENTAL RANGE, AZ US	31.76	-110.85	1310.6	[1950,2010]<br>49 valid	550 (129)
USW00023066	GRAND JUNCTION WALKER FIELD, CO US	39.13	-108.54	1470.4	[1900,2024]<br>124 valid	222 (56)
USC00047776	SAN GABRIEL CANYON PH, CA US	34.16	-117.91	226.8	[1917,2024]<br>85 valid	555 (278)

Table 1. Characteristics of three selected Southwestern weather stations; For the annual statistics, a water year is "valid" if least 95% of the days wtihin are present and not QC-flagged. Here, water year is defined to start on 1 October.

At all sites, $1/\beta$, which is roughly the mean peak-over-threshold daily precipitation accumulation, varies by season. At Santa Rita, α can be considered constant over all seasons, whereas Grand Junction and San Gabriel each show one season where α may be zero (MAM and JJA, respectively). In the case of San Gabriel, this is the dry season, when there is practically no precipitation which would surpass an annually-defined threshold. This relates back to our intuition about naively applying TED in a Mediterranean climate. In this case, the annual cycle of precipitation itself does the necessary data filtering for us. In the dry season, there are appropriately few precipitation Events identified. Given how α is estimated, small samples tend to result in the estimate of α being 0.

This dull statistical reality does not not govern Grand Junction in MAM, which shows a bona fide interesting annual cycle in α. It should be noted however, that when mixing heavy-tailed and non-heavy-tailed distributions, the result is a heavy-tailed distribution. The mixture of seasons should therefore be heavy-tailed.

Turning to event duration, we observe surprisingly little seasonality at Santa Rita. Here, roughly 85-88% of events are only one day long at any time of year. Besides SON, almost all other events are two days long. During SON, the longest events are observed. Given that this season may catch the end of the monsoon or the beginning of the winter, this may be an artefact of the threshold being low, despite a possibility for larger precipitation events.

Grand Junction's event durations have seasonality which show differences in precipitation type over the year, despite the relative lack of a seasonal cycle in monthly total precipitation. At San Gabriel, it is apparently JJA which has the highest percentage of events longer than one day. However, it has a distinct lack of events longer than two days. This indicates that there are short duration precipitation events which straddle the local midnight. If our intent is to describe characteristics of precipitation Events over the year, we might call this high proportion of super-daily Events in JJA a pathological result, since it is an artefact of the low threshold set by all of the data over JJA; more is not always better when it comes to covariates. During the rainy season, the longest precipitation events of all tend to occur in DJF, when synoptic features (midlatitude cyclones and atmospheric rivers) result in multi-day precipitation events over a wide domain.

In short, at San Gabriel, all is as we would expect, given how the parameter estimation technique works and the fact that we are looking at daily precipitation data. We are surprised that the least seasonality in parameters overall is seen at Santa Rita, where there are two distinct rainy seasons. Grand Junctions' parameters are as expected: although the seasonal cycle of precipitation is flat in terms of monthly accumulations, the characteristics of precipitation are not constant over the year, which the parameter estimates reflect. We will now see how seasonal binning affects our estimation of probabilities of event totals.

Probability (Return Interval) Estimates

We consider the probabilities and return intervals of event totals from 1 inch to 17.5 inches. We consider return intervals of 10^0 to 10^4 years to be meaningful, so we focus on this range of return intervals. Note that due the sensitivity of the concept of "event total" to the threshold used for defining events, this should be among the most sensitive quantities we can consider.

Table 2 shows return intervals of event totals for the three stations calculated by two methods. The "bulk" method used in the main text is based on a single threshold and parameter vector per location. The "seasonal" method is based on events defined on the three month seasons and parameters fit separately to each. General event-level probabilities for exceeding a given event totals are calculated as weighted averages of the probabilities of each season. The weights are the proportion of all Events which have historically occurred in each season. This is the most straightforward method for mixing distributions.

Table 2 indicates that the bulk and seasonal estimates of return intervals for very large Event totals at Santa Rita differ quite a bit. On the other hand, the estimates at San Gabriel are hardly affected (and falling squarely within the middle quartiles of the empirical distribution of the seasonal estimates). Grand Junction Falls somewhere in between. The result of San Gabriel is not surprising. In the Mediterranean climate, a fixed threshold makes us detect events precisely in the narrow season in which there is notable precipitation. In this case, we are hardly mixing distributions. The consideration of seasons by binning is akin to informally adding a covarariate, which apparently has a weak affect in this case. Nonetheless, the addition of covariates

Name	Event Total Precip [in]	Bulk RI [yr]	Seasonal RI [yr]	Bulk / Seasonal
SANTA RITA EXPERIMENTAL RANGE, AZ US	2.5	2	2	1.00
	3.0	4	3	1.33
	3.5	7	6	1.17
	4.0	15	12	1.25
	4.5	28	20	1.40
	5.0	53	34	1.56
	7.5	972	320	3.04
	10.0	12228	1945	6.29
	12.5	117661	8731	13.48
GRAND JUNCTION WALKER FIELD, CO US	1.0	2	2	1.00
	1.5	14	13	1.08
	2.0	79	68	1.16
	2.5	400	302	1.32
	3.0	1811	1181	1.53
	3.5	7451	4225	1.76
SAN GABRIEL CANYON PH, CA US	4.5	2	2	1.00
	5.0	2	2	1.00
	7.5	7	7	1.00
	10.0	23	20	1.15
	12.5	67	54	1.24
	15.0	176	142	1.24
	17.5	433	351	1.23

Table 2. Medians of bootstrapped (N=500) return intervals for precipitation Event totals exceeding various levels from 1 inch to 17.5 inches; Rows are excluded when both methods yield return levels less than 1 year or greater than 10000 years.

generally tends to reduce bias in parameter estimates. In our judgement, a reduction is bias here is probably not worth the concomitant increase in complexity of the model in variance in parameter estimates. At Santa Rita, on the other hand, the effect of ignoring the previously implicit mixture is apparent. For somebody calculating a return levels for Event total precipitation, there is a material difference on for higher Event totals.

Seasonality and Our Results

Although calculated return intervals can be affected by the choice of acknowledging or ignoring seasons, this is of little consequence with respect to what we have presented in the main text:

- **Goodness-of-Fit (Figures 4,S2):** Adding more parameters to a modeling procedure generally reduces bias. The case study on three stations suggests that return intervals for large event totals tend to be shorter when we consider seasons explicitly. In this case, making return intervals shorter (or return levels larger) is generally a correction in the correct direction.
- **15 inch return interval map (Figure 5a):** Supplemental Figure S7ab show broad agreement between empirical and parametric return intervals of Event totals exceeding 15 inches, except for the Gulf Coast, where the bulk-estimated return intervals appear to be too long. In line with the new results of this section about Mediterranean climates vs. others, we see a reflection of the same thing applying more broadly on these maps. The estimated return intervals in California agree more closely with observations than those of Washington and especially Texas. Return intervals calculated by seasonal binning and explicit mixture of distributions are less biased. For the purposes of this map, it is only the west coast and the Texan Gulf Coast where discrepancies truly matter, as 15 inch total events are exceedingly rate (perhaps impossible) in most other regions.
- **San Gabriel Case Study (Figure 5b, Tables 2,3):** This section shows that probability estimates and return intervals at San Gabriel are numerically hardly affected by seasonal binning.

Recapitulation

The TED, especially since it allows for heavy-tailed exceedances is capable of performing some implicit mixing (without any covariates) on its own. In practice, seasonality can sometimes be ignored, but not always. Until a proper (continuous)

method for treating covariates is ready (a work in progress), it makes sense to do this sort of comparison of bulk and mixture distributions whenever we apply a TED.

Floating Figures

Figure S10 is not included in the main text merely because of the allowed article length.

References

1. Wasserstein, R. L. & Lazar, N. A. The ASA statement on p-values: context, process, and purpose. *The Am. Stat.* **70**, 129–133, DOI: 10.1080/00031305.2016.1154108 (2016). https://doi.org/10.1080/00031305.2016.1154108.

2. Dowd, C. A new ecdf two-sample test statistic, DOI: https://doi.org/10.48550/arXiv.2007.01360 (2020). 2007.01360.

3. Dowd, C. *twosamples: Fast Permutation Based Two Sample Tests* (2022). R package version 1.2.0.

4. Balkema, A. A. & de Haan, L. Residual life time at great age. *The Annals Probab.* **2**, 792–804 (1974).

5. Panorska, A. K., Gershunov, A. & Kozubowski, T. J. From diversity to volatility: Probability of daily precipitation extremes. In *Nonlinear Dynamics in Geosciences*, 465–484 (Springer New York, New York, NY, 2007).

6. Cavanaugh, N. R., Gershunov, A., Panorska, A. K. & Kozubowski, T. J. The probability distribution of intense daily precipitation. *Geophys. Res. Lett.* **42**, 1560–1567, DOI: https://doi.org/10.1002/2015GL063238 (2015). https://agupubs.onlinelibrary.wiley.com/doi/pdf/10.1002/2015GL063238.

7. Baringhaus, L. & Franz, C. On a new multivariate two-sample test. *J. Multivar. Analysis* **88**, 190–206, DOI: https://doi.org/10.1016/S0047-259X(03)00079-4 (2004).

8. Franz, C. *cramer: Multivariate Nonparametric Cramer-Test for the Two-Sample-Problem* (2024). R package version 0.9-4.

9. Kremer, L. P. *ggpointdensity: A Cross Between a 2D Density Plot and a Scatter Plot* (2019). R package version 0.1.0.

List of Figures

S1 Station-by-station map of whether or not TED is appropriate to apply; a Dowd Two-sample test was conducted on both the Exceedances and Event durations, each at the $\alpha = 0.05/2$ level, with the null hypotheses being that the observations arose from the specified distributions (Pareto II and Hurdle-geometric); strictly, "Appropriate" means that neither the null hypothesis was rejected . . . 7
S2 Station-by-station map of TED goodness-of-fit; a Dowd Two-sample test[2] was conducted on the Event durations, maxima, and totals, each at the $\alpha = 0.05/3$ level, with the null hypotheses being that the observations arose from the specified distributions (the TED marginals); strictly, "adequate" means that none of the null hypotheses were rejected . . . 8
S3 A scatterplot of goodness-of-fit decisions based on simultaneous testing of marginals vs a multivariate test; the tests on the marginals are Dowd tests with $\alpha = 0.05/3$ and the multivariate test is that of Baringhaus with $\alpha = 0.05$. These tests were performed on 1/10 of the stations which had over 10 years of precipitation data. . . . 9
S4 Estimates of α and β at the stations at the first, second, and third quartiles of station record lengths. The estimate on the orignal sample is shown in red atop the 100 bootstrapped replicates shown in faint black . . . 10
S5 The mean durations on the original sample and the bootstrapped replicates from the same set of stations as shown in S4 . . . 11
S6 Semi median absolute deviations from the median of parameter estimates for 100 bootstrapped replicates of events at a random subset of 1/10 of the stations in WUS with over 10 years of observations; This is equivalent to the absolute differences between the bootstrapped 25th and 75th percentiles from the 50th percentile, as would be apparent on a boxplot. . . . 12
S7 Return intervals of 15 inch events shown 4 different ways on a random subset of 1/10 of the stations with records longer than 10 years (426 here); **a** empirical return interval calculated from the sample of events; **b** return interval calculated from the TED with maximum likelihood estimated parameters on the sample of events; **c (d)** the 5th smallest (largest) out of 100 bootsrtrapped replicates of the whole sample of events. Missing values are in grey, as in **a** when an event total exceeding 15 inches has never been observed . . . 13
S8 Point density plots[9] of all historical daily precipitation above the locally-defined 75th percentile of above-trace precipitation at Santa Rita (**a**), Grand Junction (**b**), and San Gabriel (**c**); The constant local 75th percentile is plotted as a dashed red circle for reference. The boundaries of the three-month-seasons seasons are highlighted with dark blue radial spokes. . . . 14
S9 Point density plots[9] of 500 bootstrapped parameter estimates and derived quantities for Santa Rita (**a**), Grand Junction (**b**), and San Gabriel (**c**); The estimates from the original sample are plotted as red crosses atop the point densities. . . . 15
S10 A demonstration of how most precipitation is accumulated during Events: **a)** a histogram (over the population of stations) of the proportion of all precipitation which falls during Events, which is equivalent to the proportion of precipitation which accumulates during wettest 25% of wet days; **b)** a station-point map of the average number of precipitation Events per year . . . 16

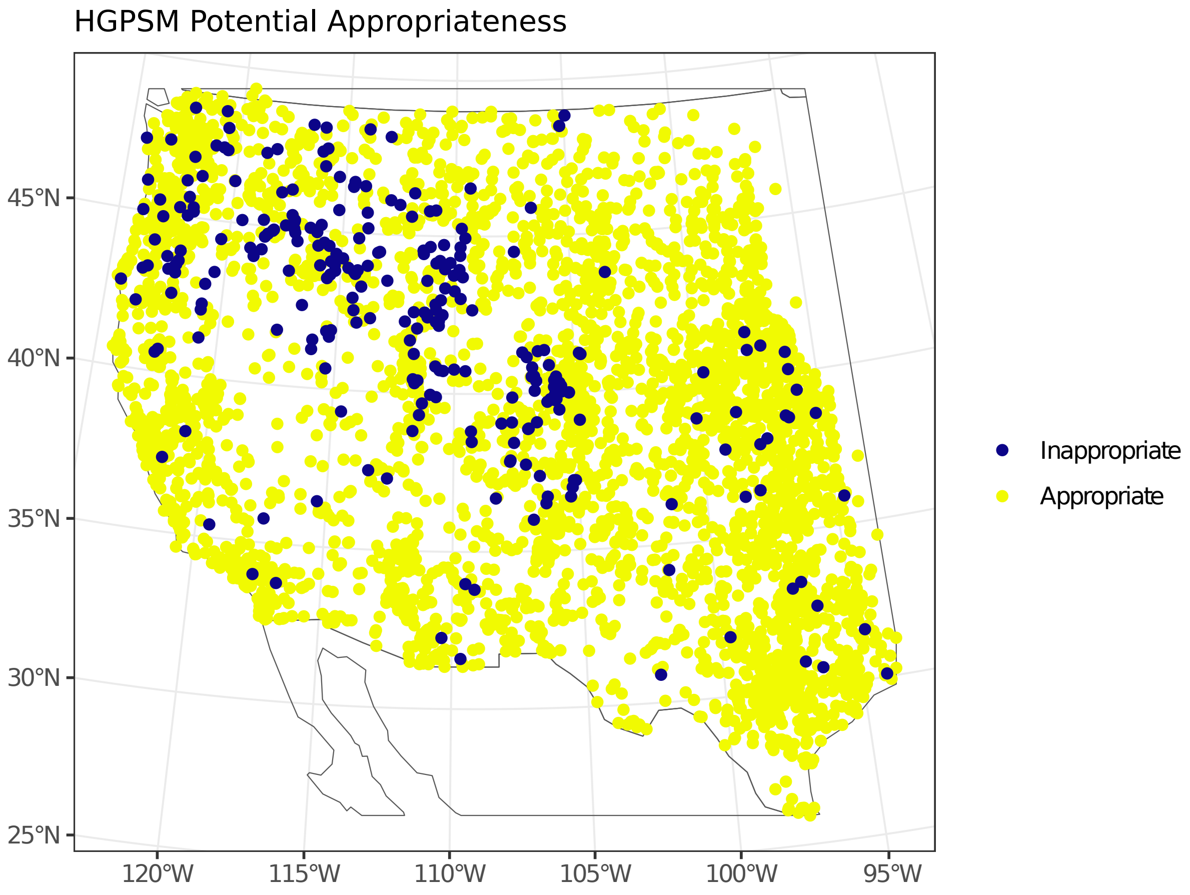

Figure S1. Station-by-station map of whether or not TED is appropriate to apply; a Dowd Two-sample test was conducted on both the Exceedances and Event durations, each at the $\alpha = 0.05/2$ level, with the null hypotheses being that the observations arose from the specified distributions (Pareto II and Hurdle-geometric); strictly, "Appropriate" means that neither the null hypothesis was rejected

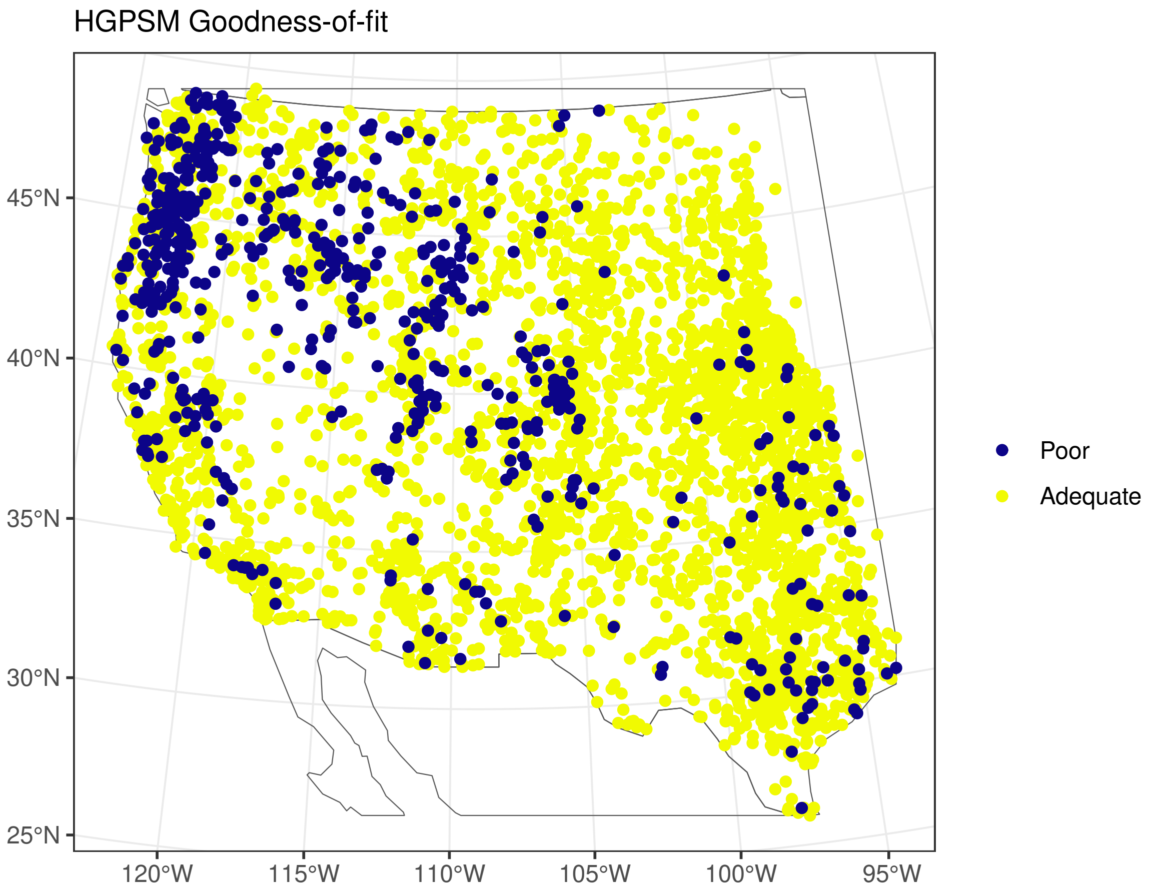

Figure S2. Station-by-station map of TED goodness-of-fit; a Dowd Two-sample test[2] was conducted on the Event durations, maxima, and totals, each at the $\alpha = 0.05/3$ level, with the null hypotheses being that the observations arose from the specified distributions (the TED marginals); strictly, "adequate" means that none of the null hypotheses were rejected

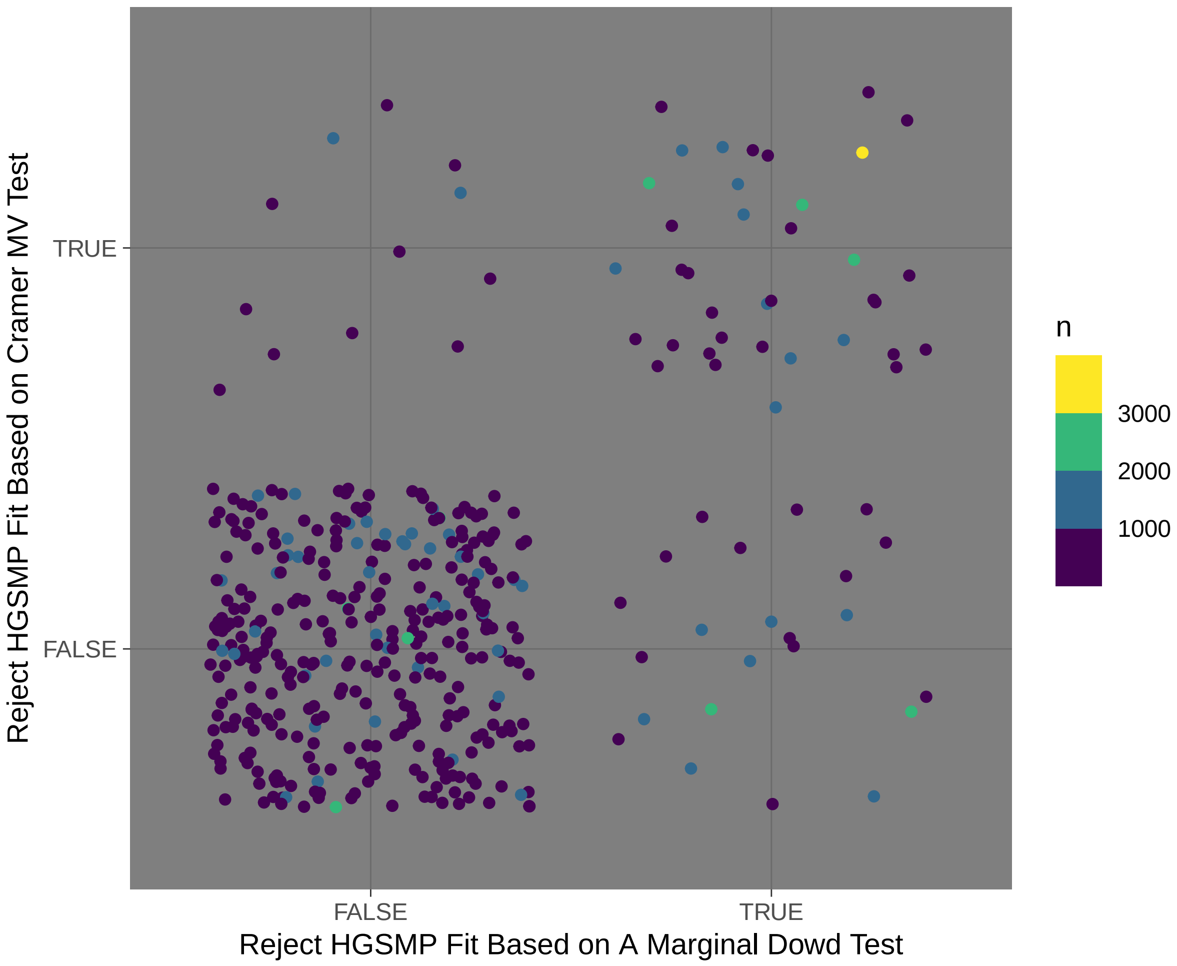

Figure S3. A scatterplot of goodness-of-fit decisions based on simultaneous testing of marginals vs a multivariate test; the tests on the marginals are Dowd tests with $\alpha = 0.05/3$ and the multivariate test is that of Baringhaus with $\alpha = 0.05$. These tests were performed on 1/10 of the stations which had over 10 years of precipitation data.

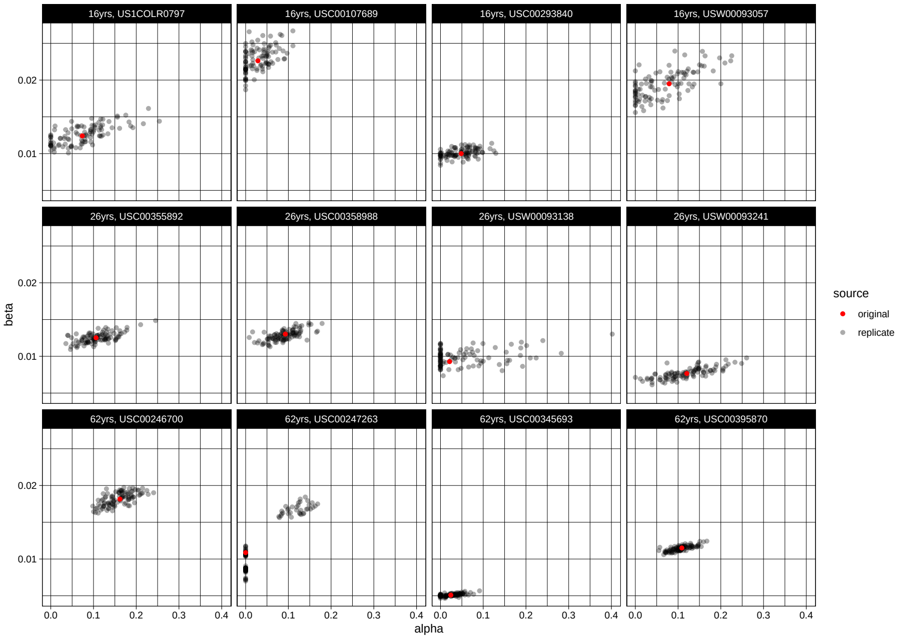

Figure S4. Estimates of α and β at the stations at the first, second, and third quartiles of station record lengths. The estimate on the orignal sample is shown in red atop the 100 bootstrapped replicates shown in faint black

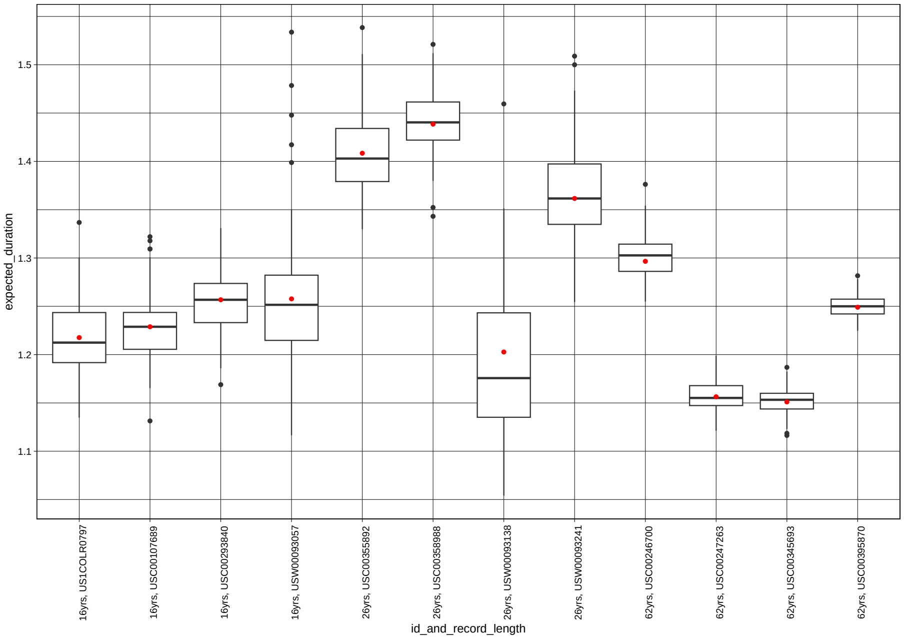

Figure S5. The mean durations on the original sample and the bootstrapped replicates from the same set of stations as shown in S4

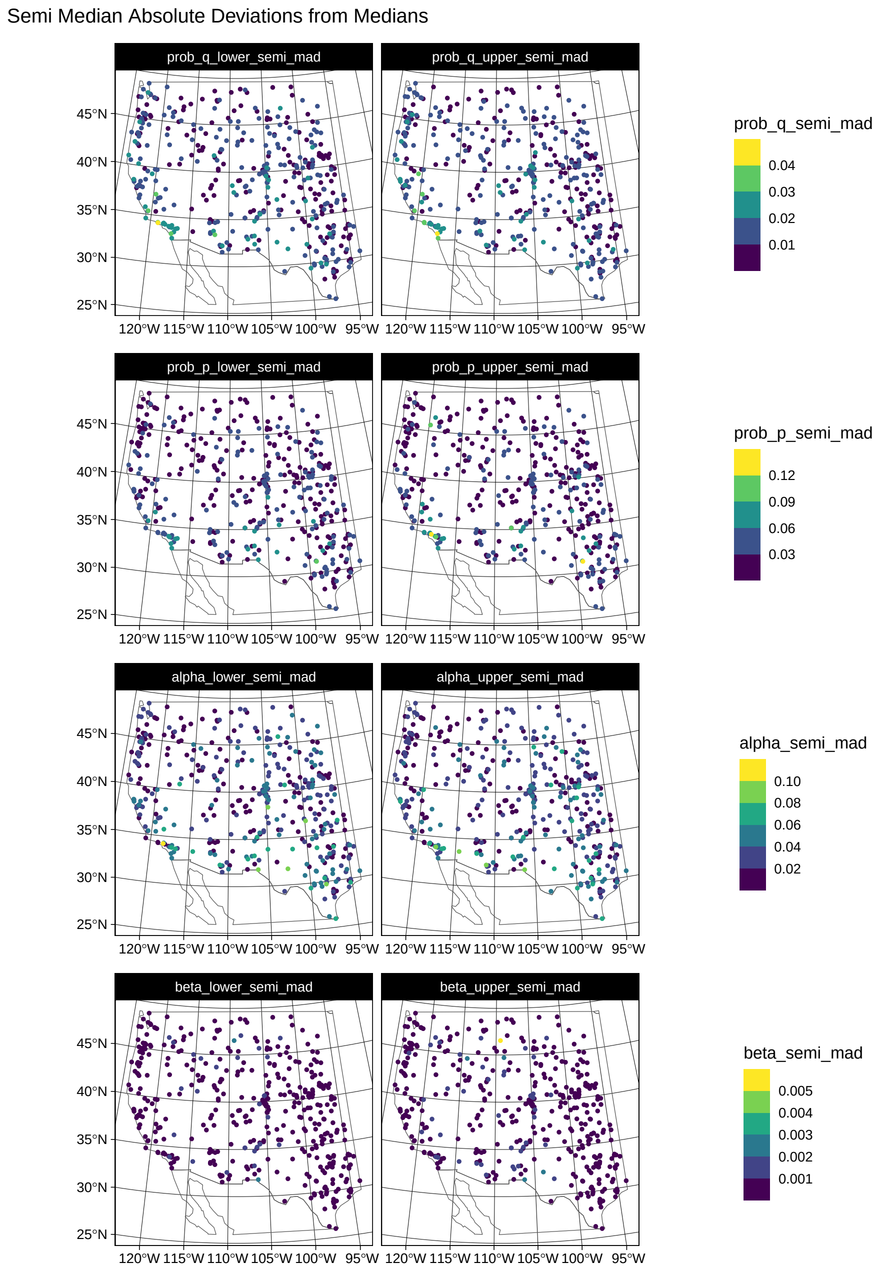

Figure S6. Semi median absolute deviations from the median of parameter estimates for 100 bootstrapped replicates of events at a random subset of 1/10 of the stations in WUS with over 10 years of observations; This is equivalent to the absolute differences between the bootstrapped 25th and 75th percentiles from the 50th percentile, as would be apparent on a boxplot.

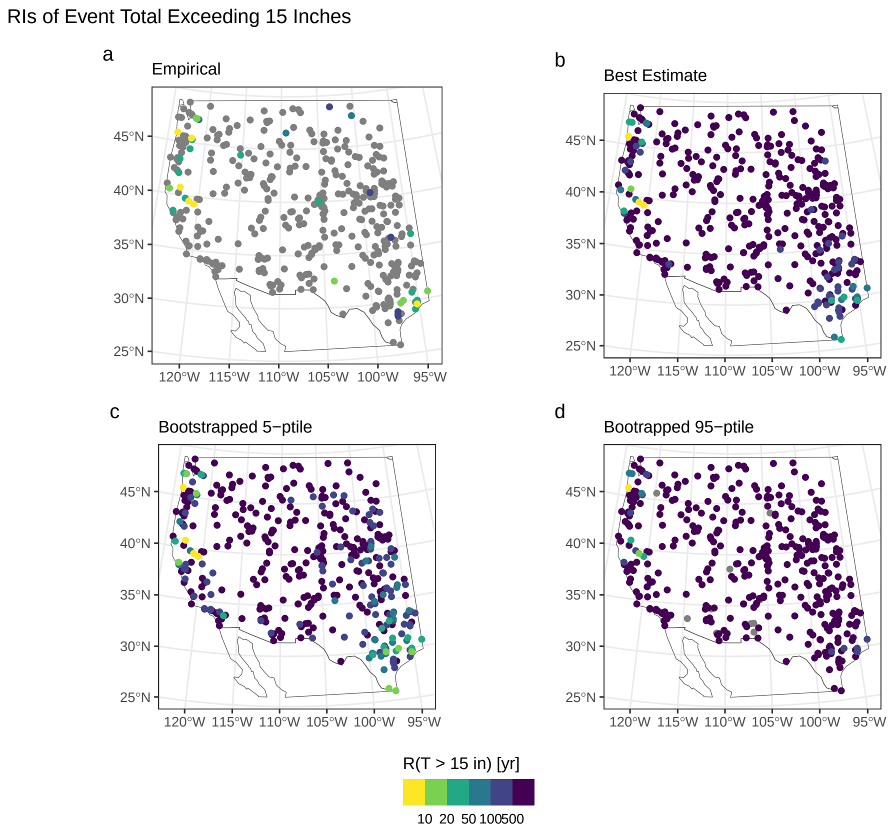

Figure S7. Return intervals of 15 inch events shown 4 different ways on a random subset of 1/10 of the stations with records longer than 10 years (426 here); **a** empirical return interval calculated from the sample of events; **b** return interval calculated from the TED with maximum likelihood estimated parameters on the sample of events; **c** **(d)** the 5th smallest (largest) out of 100 bootsrtrapped replicates of the whole sample of events. Missing values are in grey, as in **a** when an event total exceeding 15 inches has never been observed

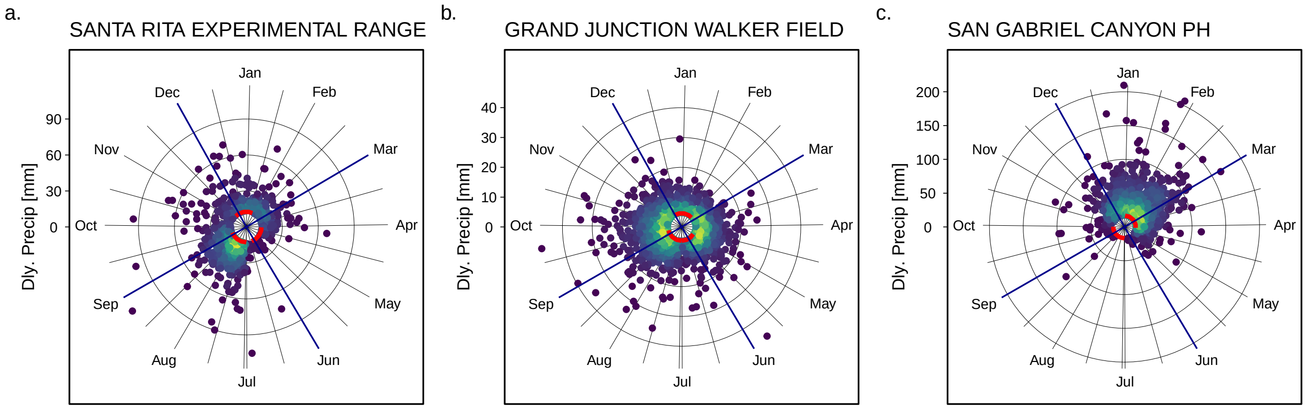

Figure S8. Point density plots[9] of all historical daily precipitation above the locally-defined 75th percentile of above-trace precipitation at Santa Rita (**a**), Grand Junction (**b**), and San Gabriel (**c**); The constant local 75th percentile is plotted as a dashed red circle for reference. The boundaries of the three-month-seasons seasons are highlighted with dark blue radial spokes.

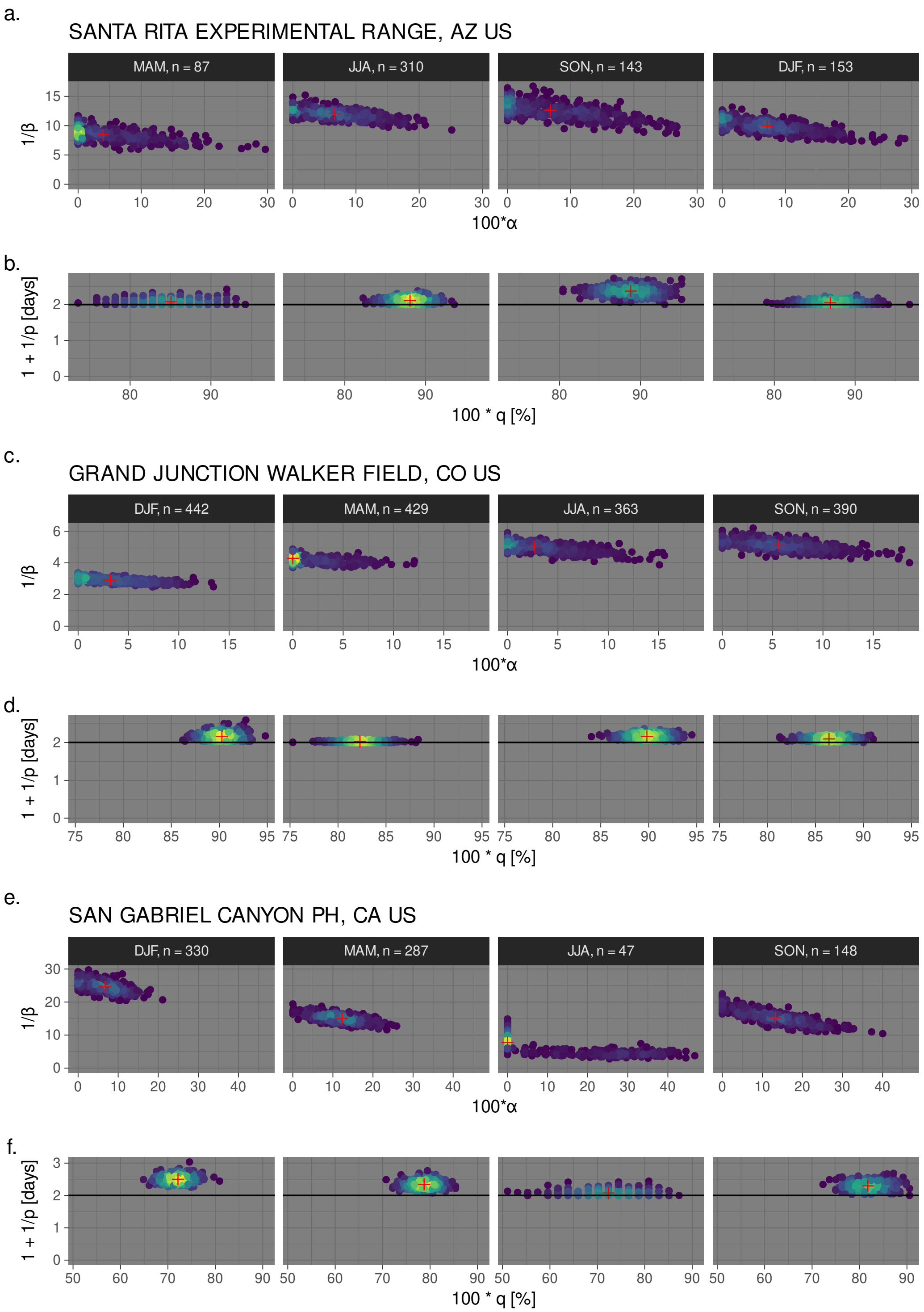

Figure S9. Point density plots[9] of 500 bootstrapped parameter estimates and derived quantities for Santa Rita (**a**), Grand Junction (**b**), and San Gabriel (**c**); The estimates from the original sample are plotted as red crosses atop the point densities.

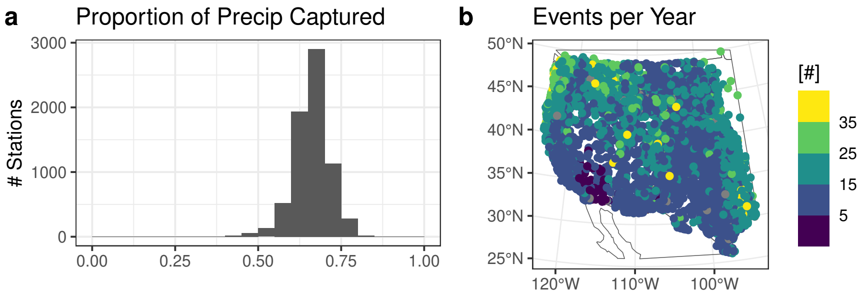

Figure S10. A demonstration of how most precipitation is accumulated during Events: **a)** a histogram (over the population of stations) of the proportion of all precipitation which falls during Events, which is equivalent to the proportion of precipitation which accumulates during wettest 25% of wet days; **b)** a station-point map of the average number of precipitation Events per year
